# Detectable, Task-Relevant, or Harmful? Construct-Valid Evaluation of Unlabeled Distribution-Shift Monitoring in Chest Radiography

**DOI:** 10.3390/jimaging12100492

**Published:** 2026-10-07

**Authors:** Dan Gabriel Badea, Răzvan-Andrei Rotaru, Răzvan Victor Rughiniș, Flavia Zaim-Oprea, Dinu Țurcanu, Daniel Rosner

**Affiliations:** 1 Faculty of Automatic Control and Computers, National University of Science and Technology POLITEHNICA Bucharest, 060042 Bucharest, Romania; razvan.rotaru1601@stud.acs.upb.ro (R.-A.R.); razvan.rughinis@upb.ro (R.V.R.); flavia.oprea@upb.ro (F.Z.-O.);; 2 Academy of Romanian Scientists, Ilfov 3, 050044 Bucharest, Romania; 3 Faculty of Electronics and Telecommunications, National Institute of Innovations in Cybersecurity “CYBERCOR”, Technical University of Moldova, 168 Ștefan cel Mare Blvd, MD-2004 Chișinău, Moldova; dinu.turcanu@utm.md

**Keywords:** dataset shift, drift monitoring, medical imaging, chest radiography, model-performance estimation, maximum mean discrepancy, error estimator, construct validity, calibration, MLOps

## Abstract

Distribution-shift alarms do not establish classifier harm. We evaluated the detectability, frozen-head relevance, and performance deterioration of two public DenseNet-121 chest-radiography classifiers under 30 controlled image conditions. Signals covered pixels, 64-component PCA representations, logits, probabilities, and calibrated high-error selection. An exact whole-model-null intervention changed representations while preserving all 18 released output slots to within 4.09 × 10^−14^; PCA monitoring detected all six interventions, five-logit monitoring detected none, and the paired Brier change was negligible. Across image shifts, PCA and logit alarm rates were 97% and 52%. In held-source/held-family prediction of primary Brier harm, probability mean displacement had the lowest error (MAE 0.00679); neither PCA signal outperformed the training-mean baseline after correction, whereas three output-facing signals beat both prespecified baselines. Secondary results were outcome-dependent: PCA RFF-MMD ranked best for macro-AUC and locked-F1 loss. An exploratory ConvNeXtV2-Atto/MobileViT-XS arm reproduced the construct separation: representation monitoring detected 57/60 conditions and all six task-null controls, while logit monitoring detected no task-null control; probability mean had the lowest held-architecture/held-family Brier-harm error ratio (0.404). The results show that detectability, task relevance, and metric-specific harm are distinct constructs and should be reported separately.

## 1. Introduction

Medical-image classifiers can encounter acquisition, population, site, and workflow changes after deployment. Cross-hospital evaluations show that performance can fall even when the task and disease definitions appear unchanged [1,2], and uncertainty and calibration can degrade under dataset shift [3]. Continuous monitoring is therefore necessary, but an alarm has an ambiguous meaning: it may indicate a change that is statistically detectable, a change that affects information used by the classifier, or a change that worsens a particular performance measure. Detectability, task relevance, and metric-specific harm are different constructs.

Two-sample tests such as maximum mean discrepancy (MMD) can detect changes in a characteristic representation [4]. They do not, by themselves, show that the frozen predictor is affected. A high-dimensional representation can move along directions ignored by its classification head. Conversely, output probabilities can remain nearly stable under changes that later cause subgroup or calibration failures. Treating all statistically significant shifts as harmful creates unnecessary alarms; treating output stability as safety can cause relevant changes to be missed.

Recent work has moved from generic detectability toward performance monitoring without immediate production labels. Podkopaev and Ramdas provide risk tracking when model errors can be observed [5]. Garg et al., Deng and Zheng, and Guillory et al. estimate the performance on unseen distributions from unlabeled statistics or confidences [6,7,8]. Amoukou et al. learn an error estimator, calibrate a high-error selector, and monitor it sequentially with false-alarm guarantees [9]. In medical imaging, Roschewitz et al. combine self-supervised representations and task outputs to identify covariate, prevalence, and mixed shifts [10]. WATCH uses weighted-conformal martingales for adaptive, diagnostically richer monitoring [11]. These advances make a simple “new detector” contribution neither credible nor necessary. The remaining methodological problem is validation: How can one test whether a monitor responds to the intended construct?

Recent medical-imaging evidence makes this validation gap concrete. A real-world chest-radiography study found that data-based monitors can detect change when aggregate model performance does not [12]. Label-free estimators now predict full confusion matrices and clinically relevant metrics across chest-radiograph domains while also exposing failure modes under controlled shifts [13]. In pathology, input-shift detection and output-confidence monitoring have likewise been reported as complementary rather than interchangeable [14]. The unresolved question addressed here is more basic: Can an evaluation prove that a statistically valid alarm is responding to task-relevant harm rather than to an inert change in the monitored representation?

The bachelor’s thesis underlying this project implemented multi-level drift monitoring and recovery for chest-radiography classifiers [15]. An accepted conference paper compares KS, PCA, MMD, LSDD, and learned-kernel detectors across two backbones and five scenarios, including cross-institutional deployment [16]. The present article does not reuse those numerical results, figures, or detector-ranking claims. It asks a different question and uses new public models, a new dataset role design, a new 30-condition panel, exact representation interventions, a learned-error comparator, and condition-level uncertainty.

The contributions are as follows:

1. An exact task-null control for the five selected outputs, supplemented by a post hoc whole-model-null scope audit that preserves every output of the complete released classifier layer in float64, and paired with a task-aligned control that imposes a prespecified logit change;
2. A construct-valid comparison of pixels, 64-component PCA representation, logits, probability, and calibrated learned-error monitoring under 30 image conditions from ten families;
3. Paired harm outcomes based on Brier score, AUC, F1, log loss, and calibration, with thresholds and calibrators locked before shifted-condition outcomes were extracted;
4. Held-source-domain and held-family validation, whose inferential unit is the shift condition, supported by family-stratified cluster bootstrap intervals;
5. Post hoc descriptive robustness audits showing that cross-checkpoint harm prediction depends on whether harm is defined by calibration, proper scoring rules, discrimination, or a locked decision threshold, and is not rescued by increasing PCA dimensionality;
6. A controlled exploratory architecture-sensitivity replication using ConvNeXtV2-Atto and the convolution-transformer hybrid MobileViT-XS, with independently trained five-label linear heads, the same image roles, the same 30 conditions, and repeated exact controls;
7. An explicit claim boundary showing what moderate cross-domain source models can establish and what requires stronger clinical validation.

## 2. Related Work

### 2.1. Dataset-Shift Detection

Concept drift includes changes over time in input distribution, label distribution, or the relationship between inputs and labels [17]. Batch dataset-shift detection instead asks whether a reference and a production sample plausibly arise from the same distribution. Rabanser et al. show empirically that power depends on shift type, representation, and dimensionality [18]. Kernel two-sample tests compare distributions through mean embeddings in a reproducing kernel Hilbert space [4]; LSDD directly estimates density differences [19]; learned kernels optimize test power [20]. These methods answer whether distributions differ, not whether the deployed model loses utility.

The previous ICSTCC study compared five such detectors in a multi-level monitoring architecture [16]. Its conclusion that MMD was a stable cross-architecture default is treated here as prior evidence, not as a premise. The present design compares mean displacement before and after a nonlinear random-feature map: both are mean-difference functionals, and the nonlinear map must demonstrate added information beyond the linear representation summary.

### 2.2. Predicting Performance Without Production Labels

Several approaches use confidence, disagreements, transport, or dataset summaries to estimate out-of-distribution accuracy [6,7,8,21]. Flühmann et al. extend this family to label-free confusion-matrix estimation and clinically relevant counting metrics in chest radiography [13]. Their assumptions can fail when a shift changes the proxy without changing errors, or changes errors without changing the proxy. Amoukou et al. address noisy error estimates by calibrating a binary high-error selector: true-error and estimated-error quantiles are chosen to maximize power subject to a false-discovery constraint, and the selected proportion is monitored sequentially [9]. We implement this calibration stage as the closest condition-level comparator, but the learned error model is weak, and the implementation should not be read as a full-strength test of their method. We do not reproduce its anytime-valid sequential confidence sequence because our independent unit is a finite shift condition rather than a streaming changepoint. Unlike metric-estimation benchmarks, our primary aim is to validate what the monitoring signal measures by pairing exact inert and task-aligned controls with condition-level harm.

### 2.3. Medical-Image Shifts and Cross-Domain Models

Chest radiography is particularly sensitive to site, acquisition, prevalence, and annotation differences [1,22,23]. CheXpert, MIMIC-CXR, and NIH ChestX-ray14 differ in population and labeling pipelines [24,25,26]. Kore et al. show empirically that image- and output-based drift signals can react differently from aggregate performance in a large real-world chest-radiograph time series [12]. Recent radiological monitoring studies have also used statistical process control for out-of-distribution surveillance [27] and an ensemble of monitors for real-time assessment of an intracranial-hemorrhage detector [28]. Roschewitz et al. demonstrate that representations and task outputs can jointly help identify real medical-image shifts [10], while current health-care monitoring guidance emphasizes that surveillance must be linked to a declared operational and clinical response [29]. Our question is complementary: If monitored spaces disagree, which construct has changed and what action is justified?

TorchXRayVision distributes reproducible source-domain models and standardized pathology mappings [30]. MedMNIST v2 provides compact, fixed biomedical benchmarks; ChestMNIST is derived from NIH ChestX-ray14 [31]. These resources allow the evaluation to be regenerated without private CheXpert weights from the thesis while preserving a realistic source-to-external-domain boundary.

## 3. Materials and Methods

### 3.1. Study Overview and Frozen Protocol

**Figure 1:**
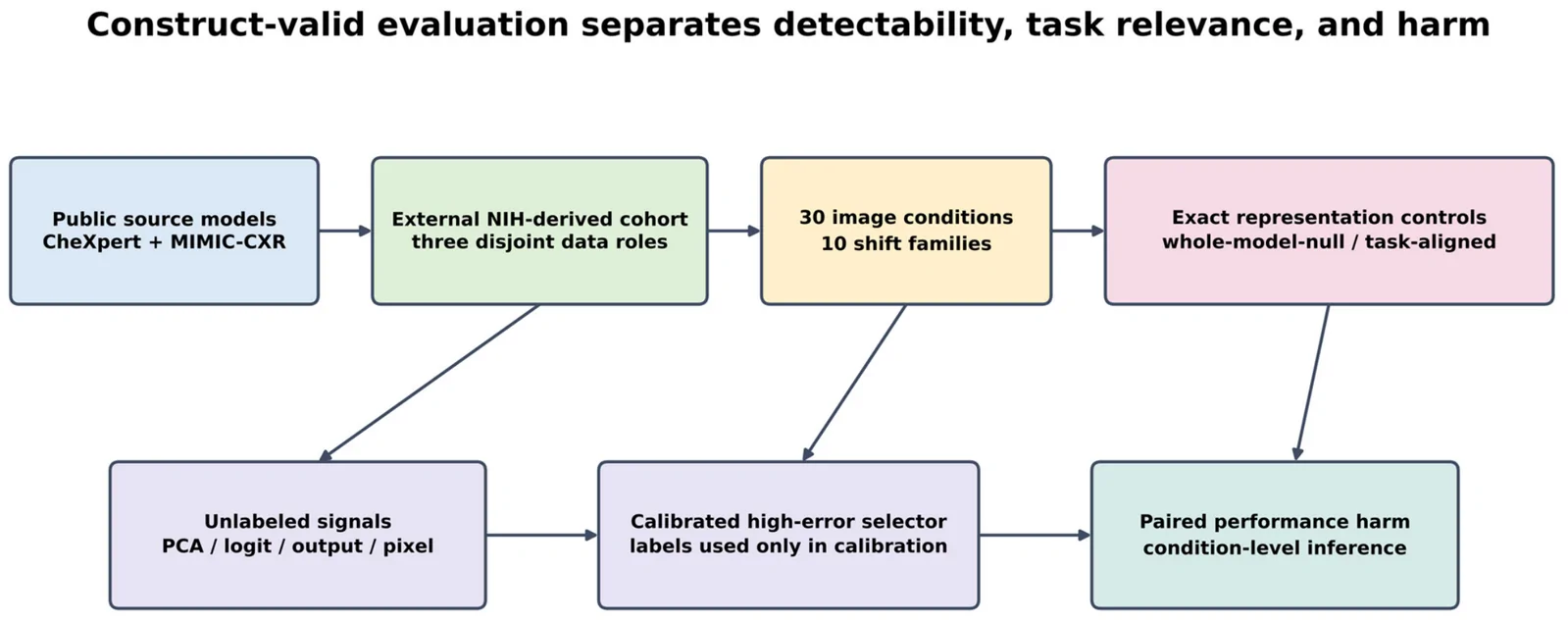
Study design. Source models and disjoint data roles feed an expanded shift panel and exact representation controls. Unlabeled monitoring signals are evaluated against paired performance harm at the condition level. Box colors only distinguish the workflow stages and carry no additional meaning.

The protocol was written before extracting any of the new shifted-image outcomes. Figure 1 separates source models, external-domain data roles, image shifts, representation controls, unlabeled signals, and delayed-label outcomes. The primary estimand is the condition-level prediction error for paired Brier deterioration. The study is methodological; “harm” means worsening of a prespecified model-performance measure.

### 3.2. Data and Source Models

We used ChestMNIST v2 at 224 pixels [31]. It contains multi-label frontal chest radiographs derived from NIH ChestX-ray14 [26]. The prespecified compact outcome panel retained five labels shared by the public source models: atelectasis, cardiomegaly, effusion, consolidation, and edema. This panel was fixed before shifted outcomes were extracted; it was chosen to keep calibration, thresholding, exact-control construction, and delayed-label outcomes on one common set rather than to maximize any observed result.

Two released TorchXRayVision DenseNet-121 checkpoints were frozen [30]: densenet121-res224-chex, trained on CheXpert, and densenet121-res224-mimic_ch, trained on MIMIC-CXR using the CheXpert label policy. This study did not retrain or fine-tune either network. Both expose a 1024-dimensional penultimate representation and an 18-slot linear classifier layer. The 18 released slots comprise the five selected outcomes: two additional active outcomes shared with ChestMNIST (pneumonia and pneumothorax); four active source outputs absent from the ChestMNIST label set (lung lesion, fracture, lung opacity, and enlarged cardiomediastinum); and seven numerically inactive rows described in Section 3.5. The five-outcome choice can affect metric-dependent results and is not presented as an exhaustive evaluation of all active outputs. The post hoc whole-model-null audit covers all 18 slots only for the invariance question. Neither checkpoint had been optimized on ChestMNIST. Their raw external-domain quality was audited on all 22,433 official test images before the drift experiment (Table 1).

The prespecified primary-model gate was macro AUC ≥ 0.75 with at least four label AUCs ≥ 0.65. Neither model passed the macro gate, and the frozen protocol specified no contingency for joint gate failure. After observing the audit values, both prespecified models were retained as sub-threshold external-domain instruments and every claim was narrowed to construct validation; this post-audit decision is disclosed in Amendment 4. No clinical deployment or safety claim is made.

### 3.3. Data Roles and Calibration

A fixed seed assigned 4000 official validation images to calibration, 2000 different validation images to drift reference, and 2000 official test images to monitoring. MedMNIST v2 documents ChestMNIST as using the official NIH ChestX-ray14 split and describes patient-level partitioning as its safeguard against leakage [26,31]. Validation-based source roles and the test-based monitor role are therefore patient-disjoint by published split provenance, although the packaged archive contains no patient identifiers with which to reverify that property locally. Calibration and reference are image-disjoint subsets of the validation split; patient-level disjointness between those two source-side roles is not established. Any repeated patients across those two source-side roles could make calibration and reference estimates more dependent or optimistic, but cannot place a monitoring image into either fitted source role under the published validation/test split. For each label, logistic calibration was fitted from the corresponding source-model logit on the calibration split. F1 thresholds were selected on those calibrated probabilities and then locked. Monitor labels were revealed only to compute outcomes after unlabeled signals had been defined.

### 3.4. Shift Panel

Thirty image conditions covered brightness, contrast, gamma, Gaussian noise, blur, rotation, resolution reduction, central occlusion, radial vignette/bias field, and fixed mixed-acquisition perturbations. Each family had three prespecified severities. The same transformed tensors were passed to both models. This panel is a controlled stress test, not a claim that each algorithmic transform reproduces a specific hospital mechanism (Table 2).

### 3.5. Exact Task-Null and Task-Aligned Controls

Let the selected frozen head be *z* = *Wh* + *b*, with representation *h* in 1024 dimensions and five logits. For a direction *v*, its task-null component is

*v_null_* = *v* − *W^T^*(*WW^T^*)^+^*Wv*,

where + denotes the Moore–Penrose inverse. Adding any scalar multiple of *v*_null_ to every representation leaves the selected logits unchanged up to numerical precision. We chose *v* as the leading reference-domain principal component before projection and used magnitudes 0.25, 0.50, and 1.00 times the median centered feature norm.

**Proposition 1** **(frozen-head invariance).** *If δ ∈ null(W), then W(h + δ) + b = Wh + b; consequently, every sigmoid probability and every deterministic thresholded decision from the selected head is unchanged. Both selected 5 × 1024 head matrices had rank 5, so their null spaces had dimension 1019. The float64 audit in* Section 4.2 *tests the numerical realization of this algebraic property.*

The frozen confirmatory construction defines task relevance relative to the five outputs analyzed in this article. A post hoc scope audit used a stricter whole-model-null control because the public TorchXRayVision classifier layer exposes 18 output slots. The same leading-PC source was projected into the null space of the complete 18 × 1024 classifier matrix. For both checkpoints, that matrix had numerical rank 11 and seven denormal-scale classifier rows (Infiltration, Emphysema, Fibrosis, Pleural Thickening, Nodule, Mass, Hernia), whose largest float64 row norm was 2.58 × 10^−38^; these rows were numerically inactive at operating precision. The resulting null space had dimension 1013. This post hoc control preserves every released output slot, not only the five outcomes used for harm estimation. It used dedicated random-number streams and did not alter any frozen condition, control, bootstrap, or CSV result.

For the task-aligned control, a desired five-logit offset, *d*, was realized by the minimum-norm feature perturbation

Δ*h* = *W^T^*(*WW^T^*)^+^*d*.

All components of *d* were fixed to 0.25, 0.50, or 1.00. Labels were not used to choose direction or magnitude. Because the same constant is added to every image for a given label, this intervention preserves within-label score ordering and leaves each label AUC—and therefore macro AUC—unchanged by construction. It probes output displacement, calibration, and locked-threshold relevance, not discrimination harm.

The exact controls are paired interventions on the same 2000 monitor images. Their alarm permutations compare each altered monitor representation with its clean-monitor counterpart, thereby isolating the intervention from the validation-to-test sampling boundary. Deployment-style image conditions instead compare the prespecified validation reference with the transformed monitor sample.

### 3.6. Monitoring Signals

The 1024-dimensional penultimate representation was reduced to 64 whitened principal components estimated from all 2000 reference images; we therefore call this arm PCA-representation monitoring, not full-representation monitoring. Each alarm test then sampled 200 reference images from that same fitted pool, leaving residual fit–test dependence; the within-reference and across-split clean controls in Section 4.3 empirically check the complete pipeline. MMD was approximated with 256 random Fourier features (RFFs), whose bandwidth used a reference-only median-distance heuristic [32]. The reported RFF-MMD is the squared distance between mean RFF vectors, ‖mean(φ(X)) − mean(φ(Y))‖^2^: a biased finite-feature V-statistic approximation to MMD^2^, not the unbiased U-statistic estimator. Thus, simple displacement and RFF-MMD apply the same mean-difference functional under linear and nonlinear feature maps, respectively. Logit and probability variants used the five selected outputs. A raw-image baseline used a fixed Rademacher projection from 14 × 14 area-downsampled pixels to 64 dimensions.

A post hoc descriptive exact-head mechanism audit used the already cached paired clean and transformed features. With *P_W_* = *W*^T^(*WW*^T^)^+^*W*, each per-image feature change Δ*h_i_* was decomposed into task-row and task-null components. The row space contains precisely the feature directions that can alter the five selected logits through the frozen linear head; the orthogonal null space is annihilated by that head. For every condition we reported Σ*_i_*‖*P_W_*Δ*h_i_*‖^2^/Σ*_i_*‖Δ*h_i_*‖^2^ and separately decomposed the paired mean displacement. For an orientation-free isotropic displacement in 1024 dimensions, the expected squared-energy share in this rank-5 row space is 5/1024 = 0.00488; enrichment ratios use this descriptive geometric benchmark. These Euclidean energy fractions are geometric summaries, not MMD values, causal attributions, or fractions of performance harm. They were not included among the nine prespecified candidate signals or multiplicity tests. The exact released classifier weights were used because recovering the head from finite cached feature-logit pairs is ill-conditioned.

The closest harmful-shift comparator followed Amoukou et al. [9]. Half of the calibration split trained a gradient-boosting regressor [33] to predict each image’s mean five-label Brier error from logits, probabilities, entropy, and margins. The other half searched true-error quantiles 0.50–0.95 and predicted-error quantiles 0.10–0.90. The pair with maximum selector power subject to false-discovery proportion ≤ 0.20 was frozen. We evaluated both the change in selected high-error proportion and the change in mean predicted error; frozen selector characteristics are shown in Table 3.

### 3.7. Alarm Calibration

PCA-representation and logit RFF-MMD were the two prespecified alarm tests. For each condition, the observed statistic was computed on a fixed 200-case reference window and a fixed 200-case monitor window. The 400 pooled observations were randomly reassigned to two groups of 200 on each of the 999 permutations; an exceedance was any permuted statistic greater than or equal to the observed statistic. The finite-sample Monte Carlo *p*-value was (1 + b)/(1 + 999), where b is the exceedance count, and the minimum attainable value was 0.001. Exact controls used clean and altered monitor pools. Clean-versus-clean type-I behavior used 30 independently resampled 200/200 reference windows per model. A separate post hoc descriptive diagnostic repeated 30 clean tests across the actual deployment boundary—validation-split reference versus clean test-split monitor—using the already fitted PCA and RFF maps and a dedicated RNG that did not alter the frozen analysis stream.

### 3.8. Outcomes and Inference

The primary outcome was the paired change in mean five-label Brier score [34] between transformed and clean predictions on the same monitor images. Secondary outcomes were changes in macro AUC, locked-threshold macro F1, log loss, and expected calibration error [35]. Because these outcomes measure different constructs, “harm” is metric-specific: positive harm denotes increasing Brier score, log loss, or ECE, and decreasing macro AUC or locked-threshold macro F1.

For every signal, a one-variable ridge mapping was trained on nine image-shift families from one source-training domain and tested on the excluded family in the other source-training domain, then reversed. The 20 folds therefore held out both source domain/model instance and family. The training-family mean harm and the ordinal low/medium/high severity rank were prespecified baselines. Five thousand bootstrap replicates resampled condition clusters with replacement within the family while retaining the paired model observations. The 60 model-condition rows were not treated as 60 independent units: the condition cluster—not the 2000 images, the two model observations, or the 999 permutations—was the inferential unit. For each signal–baseline comparison, the two-sided empirical *p*-value was twice the smaller tail proportion of bootstrap paired-MAE differences on either side of zero, with a one-count correction and truncation at 1. Empirical *p*-values covered all nine signals against both baselines (18 comparisons) and were adjusted by Holm’s step-down procedure [36]. With 5000 replicates, the minimum attainable two-sided empirical *p*-value was 2/5001 = 0.0003999; values at that resolution floor, and the corresponding Holm bounds, are reported with “≤”. A superiority claim required adjusted *p* < 0.05 against both baselines; other point rankings are descriptive.

The frozen transfer mapping and multiplicity-controlled superiority analysis targeted primary Brier harm. As a post hoc robustness audit, we applied the identical held-source-domain/held-family mapping separately to each of the four prespecified secondary outcomes. For comparability across outcome scales, Section 4.6 reports each signal’s held-family MAE divided by the corresponding training-family mean-harm MAE; values below 1 indicate lower error. No additional *p*-values or superiority claims were produced. Pearson R^2^ and Spearman correlations between each signal and positively oriented harm are descriptive associations, not evidence of a mechanism.

#### 3.8.1. Post Hoc Sensitivity and Resource Profiling

To assess whether the principal findings depended on a single PCA dimension, alarm threshold, severity stratum, or signal type, a descriptive audit repeated the PCA analysis with 16, 32, 64, 128, and 256 retained components using dedicated fixed RFF maps and 499 permutations. It also summarized the original 999-permutation alarm decisions at *α* = 0.01, 0.05, and 0.10 and separately within the prespecified low, medium, and high severity levels. These audits were not used to redefine the confirmatory analysis or generate new superiority claims.

Incremental computation was profiled for one 200-case monitoring window after model outputs and representations were available. Each signal was warmed up 15 times and timed over 200 repetitions on the analysis workstation. Persistent state includes fitted projection, RFF, or error-model parameters; working memory is an approximate array footprint for one window. Image decoding and CNN inference were excluded because they are shared upstream costs and strongly hardware-dependent.

Implementation used Python 3.12.10, PyTorch 2.12.1, TorchXRayVision 1.5.4, NumPy 1.26.4, SciPy 1.17.1, and scikit-learn 1.9.0 [37]. Seeds, model hashes, image-role indices, condition caches, and scripts are recorded in the supplementary artifact.

#### 3.8.2. Exploratory Architecture-Sensitivity Analysis

To address whether the construct findings depended on DenseNet-121, we froze a separate exploratory protocol before downloading the additional weights or analyzing their outcomes. The arm used ImageNet-pretrained ConvNeXtV2-Atto, a modern pure convolutional model [38], and MobileViT-XS, a lightweight convolution-transformer hybrid [39], through the timm 1.0.28 implementation [40]. Each encoder was frozen. Five independent L2-regularized logistic heads (C = 1, lbfgs, maximum 2000 iterations) were fitted on global-pooled representations from all 78,468 official ChestMNIST training images. Thus, neither the validation role nor the test monitor cohort trained the task heads.

The original validation/test indices were retained: 4000 validation cases for univariate probability calibration and locked F1 thresholds, a disjoint 2000-case validation reference, and the same 2000-case test monitor. The original five labels, 30 image conditions, signal definitions, 999-permutation alarms, paired outcomes, and error-proxy procedure were repeated. ConvNeXtV2-Atto used a 320-dimensional 224 × 224 representation; MobileViT-XS used a 384-dimensional 256 × 256 representation after deterministic bicubic resizing. The task heads provided explicit 5 × d maps, allowing the same exact task-null and task-aligned controls at magnitudes 0.25, 0.50, and 1.00.

The exploratory quality gate required clean-monitor macro AUC ≥ 0.70 and at least four of five label AUCs ≥ 0.65 for each model. The matched design was intentional: holding the cohort, label panel, data roles, head class and training procedure, perturbations, and inferential pipeline fixed made the encoder family the principal changed design factor. This tests architecture sensitivity without mixing the architecture comparison with a different task, dataset, or shift panel. Bidirectional transfer trained a one-signal ridge mapping on nine shift families from one architecture and tested the held family in the other architecture, then reversed, giving 20 descriptive folds per signal. We report MAE relative to the training-family mean-harm baseline and folds improved, but make no new multiplicity-adjusted superiority claim. The complete frozen protocol, per-label quality, controls, condition rows, and transfer folds are in Supplementary Section S14.4 and the reproducibility archive.

### 3.9. Use of Generative Artificial Intelligence

OpenAI Codex (GPT-5, OpenAI, San Francisco, CA, USA; accessed on 24–27 August 2026) and Anthropic Claude Opus 5 (Anthropic, San Francisco, CA, USA; accessed on 24–27 August 2026) were used for language proofreading and styling.

## 4. Results

### 4.1. External-Domain Model Audit and Calibrated Monitor Quality

**Figure 2:**
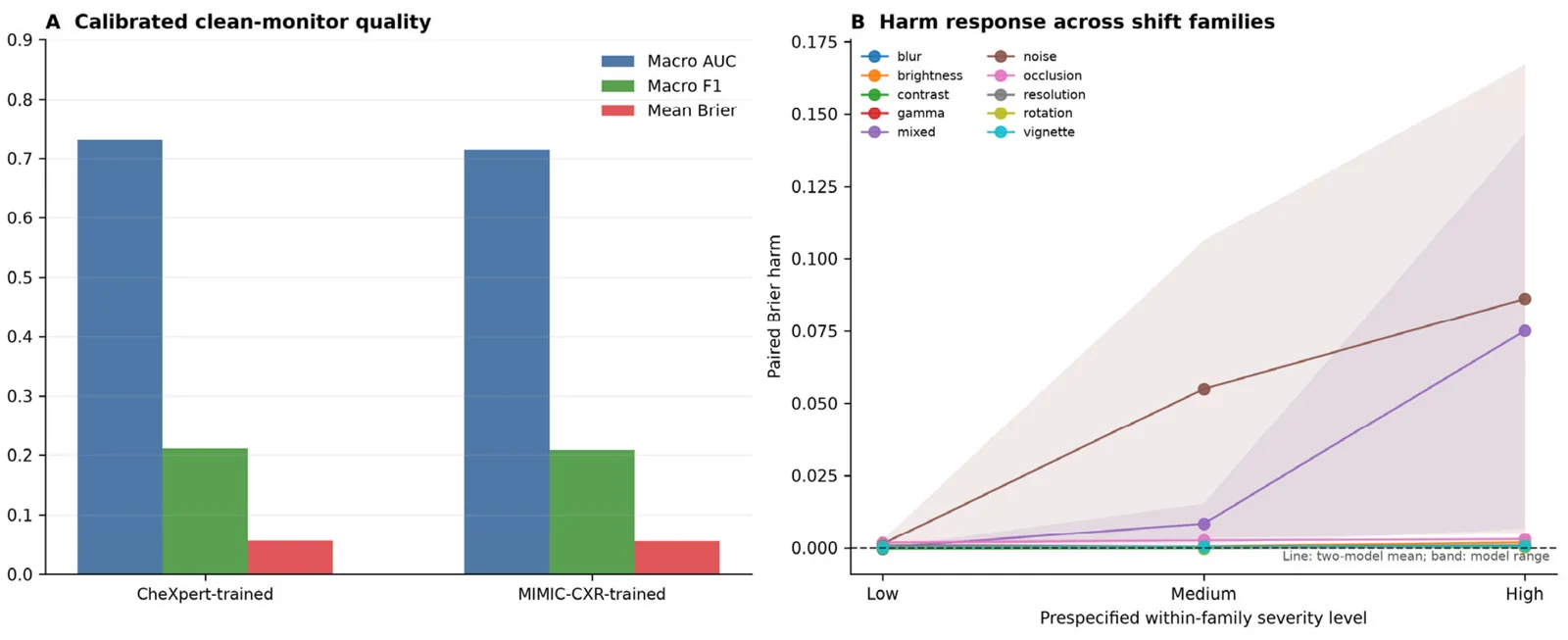
Clean-monitor model quality and paired Brier response across ten shift families. Panel (**A**) shows calibrated clean-monitor macro AUC, macro F1, and mean Brier score for the CheXpert-trained and MIMIC-CXR-trained models. Panel (**B**) uses prespecified ordinal severity levels; lines are two-model means and shaded bands, drawn in the corresponding family color at reduced opacity, span the two source models. Families with near-zero harm overlap along the dashed zero-harm line.

The full test audit yielded macro AUC 0.733 for the CheXpert-trained model and 0.714 for the MIMIC-CXR-trained model. These values quantify the intended cross-domain challenge and fell below the frozen 0.75 primary gate. Because the protocol had no joint-failure contingency, retention as a sub-threshold construct-validation instrument is a disclosed post-audit amendment rather than a prespecified eligibility outcome. Calibration on a disjoint validation role improved the interpretation of probabilities and locked the operating points used for harm outcomes; it did not change the AUC (Table 4 and Figure 2A). Figure 2B shows the paired Brier response across the ten shift families and three severity levels.

### 4.2. Exact Controls Separate Detectability from Harm

**Figure 3:**
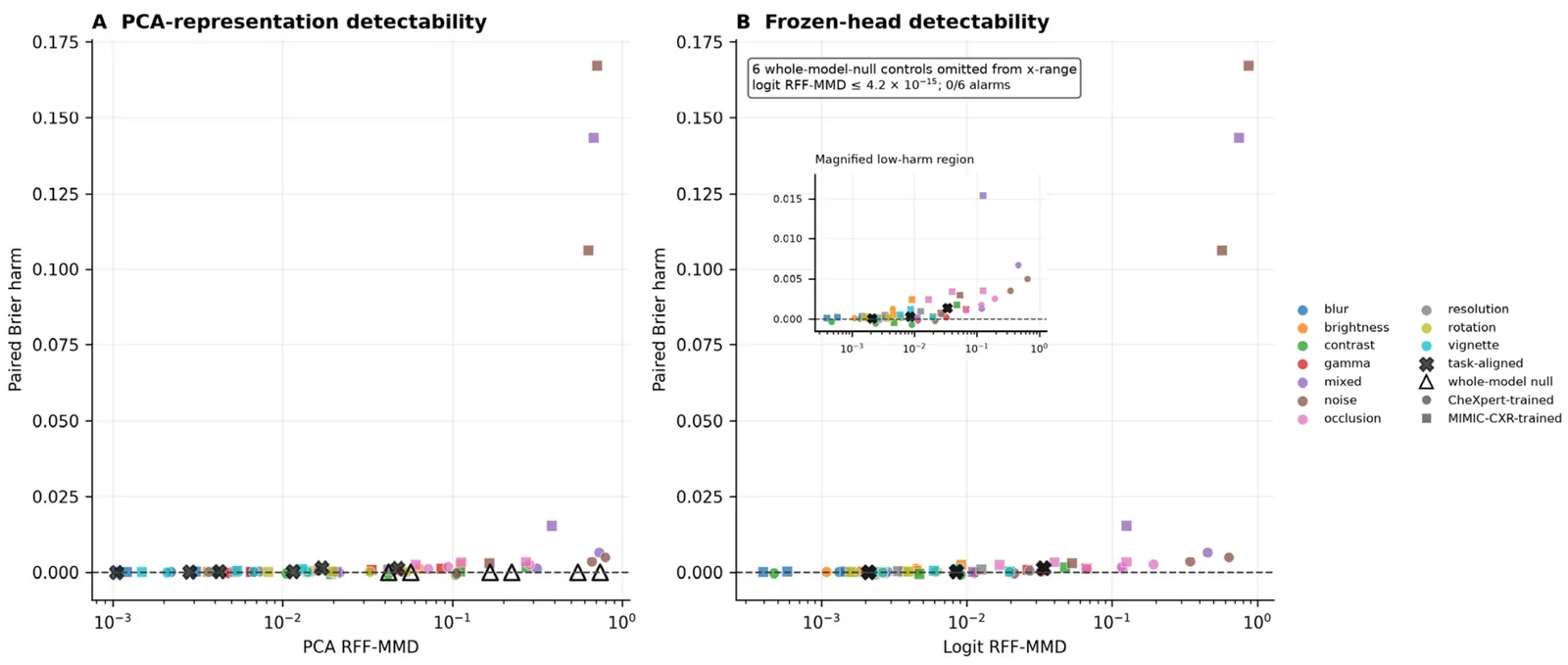
Detectability versus paired Brier harm. Hollow triangles in Panel (**A**) are whole-model-null interventions; Panel (**B**) omits their float64-zero five-logit MMD values from the displayed x-range and reports them in an annotation. Black crosses are task-aligned interventions. Image conditions are colored by family; circles and squares denote the CheXpert- and MIMIC-CXR-trained models. Dashed horizontal lines mark zero paired Brier harm, and the inset in Panel (**B**) magnifies the low-harm region.

Across six model-by-magnitude whole-model-null controls, the maximum absolute float64 change over all 18 output slots was 4.086 × 10^−14^; the maximum absolute paired Brier change was 9.872 × 10^−11^. PCA-representation MMD detected 100% of these deliberately inert conditions, while five-logit MMD detected 0%. The complete released classifier matrices had rank 11 and therefore 1013-dimensional null spaces. Thus a valid distribution detector can correctly report representation change even when every output of the released linear classifier layer is invariant (Figure 3). The original frozen five-output-null control is retained as a narrower, prespecified control; its excluded-output scope is quantified in Supplementary Section S10.

The whole-model-null PCA-representation RFF-MMD values ranged from 0.0421 to 0.7416, within the pooled image-condition range of 0.0011 to 0.7962. Within each source model, their empirical ranks spanned the 60th to 97th percentiles of the 30 image conditions. Detection therefore was not confined to interventions outside the observed image-shift scale. A separate direction audit of the frozen five-output construction rejected the concern that its leading-PC source was uniquely favorable: all 30 tests along PC 1, 8, 32, 64, or a randomly selected-output-null direction yielded *p* = 0.001. Conversely, an exact selected-output-null direction, constrained outside the retained 64-PC span, produced no alarms (0/6; *p* = 0.559–0.866), exposing the truncation blind spot by construction. The complete audits are reported in Supplementary Sections S4 and S10.

The common-offset task-aligned controls produced full- and logit-space detection rates of 83% and 50%. Their logit changes were realized to the recorded float64 tolerance without using labels to choose direction. Paired Brier changes ranged from 0.00004 to 0.00135, whereas the macro-AUC change was exactly zero in all six rows (maximum absolute change 0), as required by the rank-preserving construction. These controls therefore demonstrate detectability of deliberate output displacement, not controlled discrimination harm; complete outcomes are in Supplementary Section S4.

### 4.3. Alarm Behavior Across Image-Shift Families

**Figure 4:**
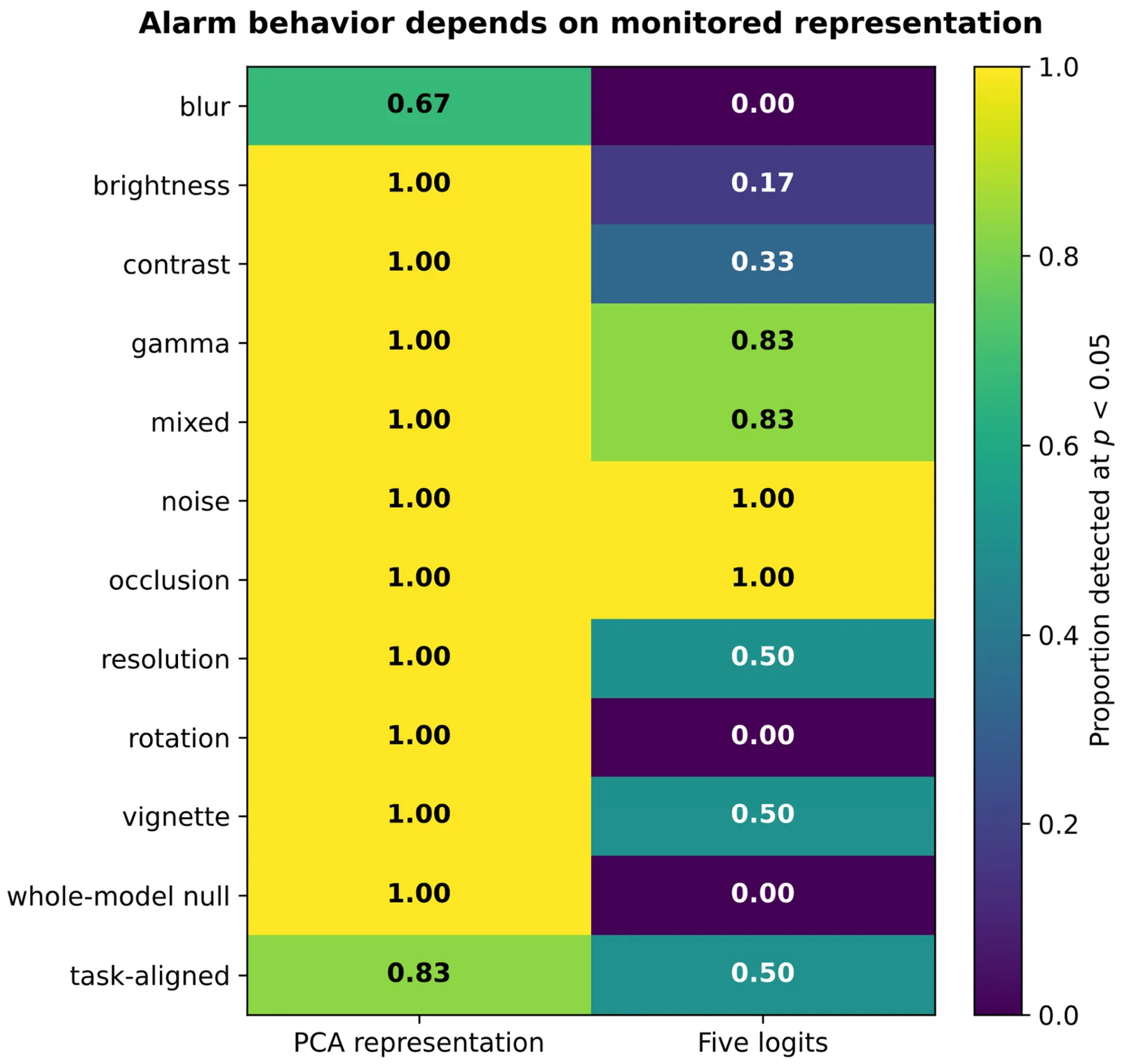
Proportion of conditions detected at *p* < 0.05 by shift family and monitored representation. Each image-family cell aggregates three severities and two source models.

Across the 60 paired model-condition observations representing 30 condition clusters, PCA-representation and logit monitoring detected 97% and 52%, respectively. These deployment-style tests compare the validation reference with the transformed monitor sample and are not numerically commensurable with the paired same-cohort exact-control rates. The difference varied by family (Figure 4), demonstrating that monitored geometry determines which changes are visible. Clean-versus-clean false-positive rates were 0.03 (1/30 windows) and 0.07 (2/30) for PCA-representation monitoring, and 0.03 (1/30) and 0.07 (2/30) for logit monitoring.

The five selected reference logits were strongly concentrated: their effective ranks were 1.13 and 1.23 of 5, with 94.1% and 90.1% of variance on the first logit principal component. This documents the low-dimensional geometry seen by the five-logit monitor, but it does not by itself explain the alarm-rate gap; permutation-test power also depends on standardized effect size, kernel bandwidth, and covariance.

The post hoc across-split clean diagnostic matched the comparison used for the image conditions. CheXpert-trained PCA/logit monitoring alarmed in 2/30 and 2/30 windows; MIMIC-CXR-trained PCA/logit monitoring alarmed in 1/30 and 2/30. Exact 95% binomial intervals were [0.008, 0.221] for 2/30 and [0.001, 0.172] for 1/30. Median *p*-values across the four cells ranged from 0.363 to 0.535. This descriptive diagnostic provides no evidence of a systematic split-boundary alarm and makes the boundary alone unlikely to explain the 97%/52% shifted-condition rates.

### 4.4. Task-Row Energy of Paired Image-Condition Changes

The post hoc exact-head audit connected the constructed invariance control to the 30 image conditions. At the median condition, 12.6–13.5% of squared paired per-image feature-change energy lay in the five-dimensional task row space. This was 25.8–27.7 times the orientation-free expectation of 5/1024 (0.488%), showing substantial enrichment toward the task row space (Table 5). Simultaneously, 86.5–87.4% lay in the 1019-dimensional null space and made exactly zero linear contribution to the five selected logit changes. Paired mean displacements had task-row fractions of 0.078 and 0.117, or 16.0× and 24.1× the same benchmark. These descriptive fractions quantify feature geometry, not the fraction of harm explained, and were computed after the confirmatory analysis.

### 4.5. Unlabeled Prediction of Primary Brier Harm

**Figure 5:**
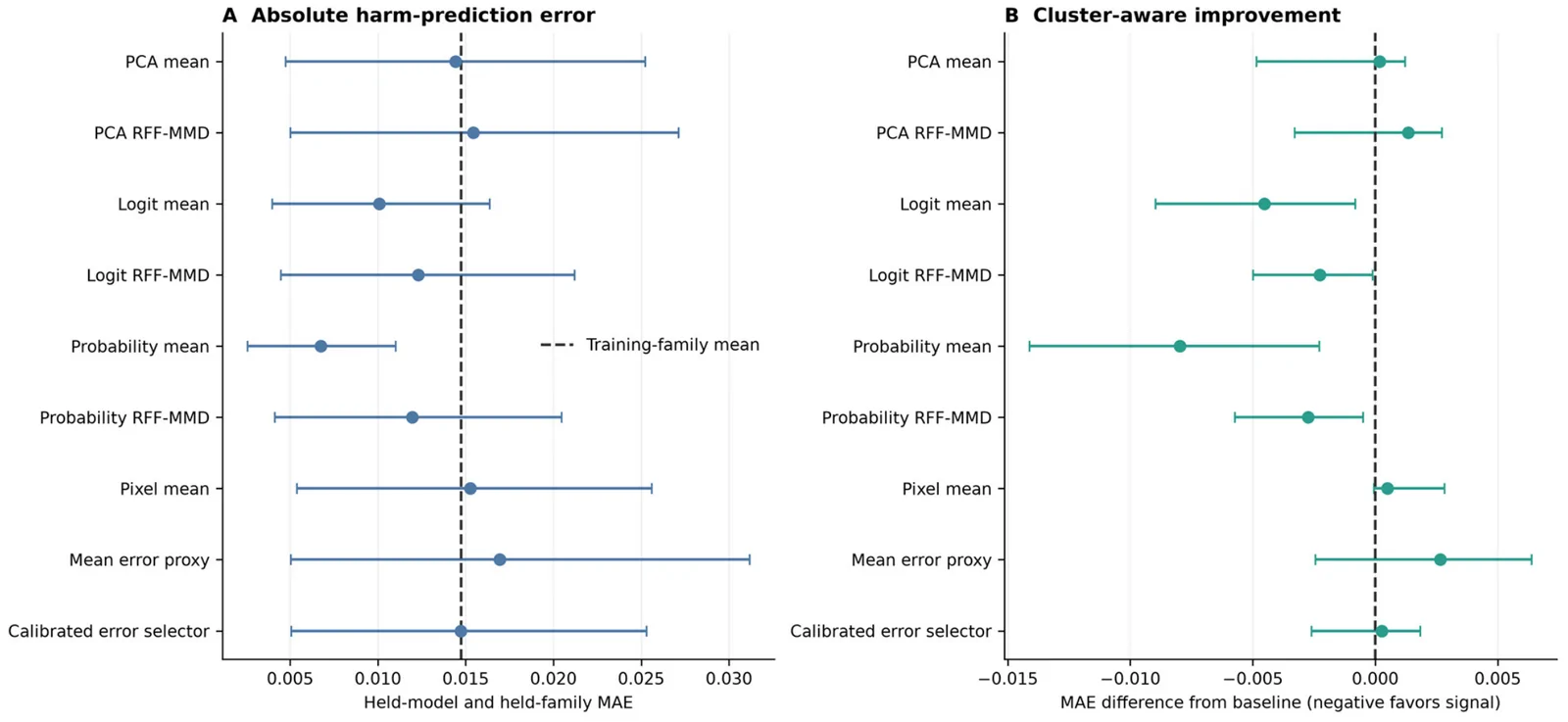
Held-source-domain and held-family prediction of primary Brier harm. Panel (**A**) reports cluster-bootstrap absolute MAE; Panel (**B**) reports paired differences from the training-family mean reference. Both source-domain model instances use DenseNet-121.

The lowest point MAE belonged to probability mean displacement (0.00679); the training-family mean and severity-rank baselines were 0.01478 and 0.01483. The cluster-bootstrap median absolute MAE interval for this signal was [0.00257, 0.01101], compared with [0.00480, 0.02494] for the training-mean baseline and [0.00490, 0.02647] for severity rank. After Holm correction across all 18 comparisons, the signals superior to both baselines were Logit mean displacement, Probability mean displacement, and Probability RFF-MMD (Table 6 and Figure 5).

Condition-level Spearman correlations with paired Brier harm ranged from 0.217 to 0.742. These associations are descriptive because conditions within a family are ordered severities, not independent samples from a natural deployment population.

### 4.6. Outcome Dependence of Held-Family Transfer

The secondary-outcome audit changed the point ranking (Table 7). Probability mean displacement had the lowest MAE ratio for Brier (0.459), log loss (0.329), and ECE (0.301). PCA-representation RFF-MMD had the lowest ratios for macro-AUC loss (0.570) and locked-F1 loss (0.609). Conversely, probability mean displacement exceeded the training-mean error for macro-AUC and locked-F1 loss, with ratios 1.630 and 1.740. Thus, no signal was an outcome-invariant point winner.

ECE did not worsen under every transformation: it increased in 38/60 model-condition rows and decreased (improved) in 22/60. The positively oriented ECE-harm analysis retains these signed changes rather than treating every detectable shift as deterioration.

This reversal was not simply a loss of condition ordering. Probability mean displacement retained positive moderate-to-strong Spearman associations with positively oriented harm across all five outcomes (0.517–0.788; Supplementary Section S7), while its calibrated held-family magnitudes were poor for AUC and F1. Pearson R^2^ likewise varied by signal and outcome: for probability mean displacement, it was 0.969 with Brier harm and 0.399 with AUC loss; for PCA-representation mean displacement, the corresponding values were 0.513 and 0.661. The high probability–Brier association is consistent with functional overlap because both are computed from model probabilities, but it is not a purely algebraic identity: Brier change also depends on labels and residual cross-terms.

A post hoc sensitivity analysis removed the frozen per-label calibrators from both probability mean displacement and Brier harm by using raw sigmoid outputs. The held-family MAE ratio remained similar (0.440 without calibration versus 0.459 with calibration), whereas Spearman correlation fell from 0.742 to 0.367 and Pearson R^2^ changed from 0.969 to 0.957 (Supplementary Table S7.4). Thus, the held-family transfer advantage was robust to removing the fitted calibration map, but the descriptive rank association was sensitive to the processing pipeline.

### 4.7. Learned-Error Comparator

The learned error regressors were weak: cross-validated R^2^ values were 0.058 and 0.081, so they explained little per-image error variance. Calibration found selectors satisfying the prespecified FDP constraint for both source models. The selector tests the calibration principle of Amoukou et al. [9], but its poor condition-level standing is not evidence against their method: our small-split regressor is not a strong proxy, and we do not reproduce the anytime-valid sequential confidence sequence. We therefore interpret this implementation only as a weak calibrated batch analogue. A fair comparative test requires a separately validated error regressor meeting a prespecified quality floor.

### 4.8. Sensitivity and Incremental Resource Profile

**Figure 6:**
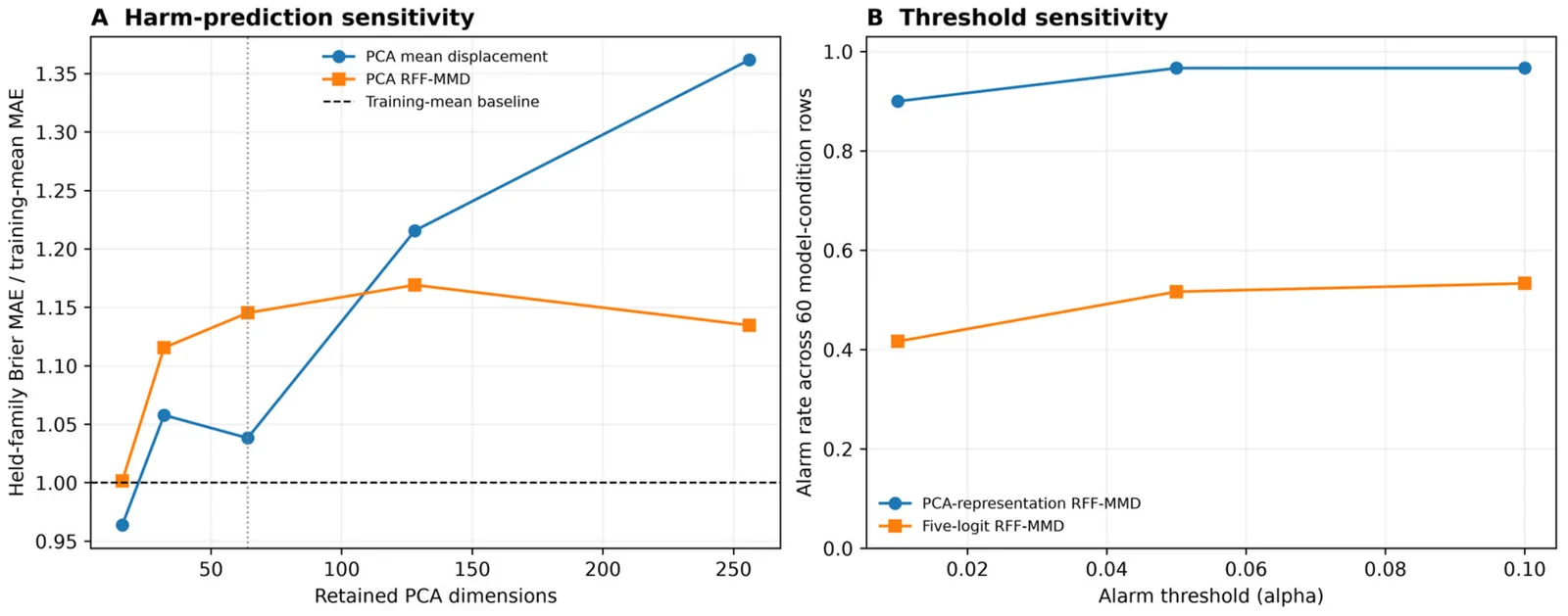
Post hoc sensitivity analysis. Panel (**A**) reports held-source-domain/held-family Brier-harm MAE relative to the training-family mean baseline as the retained PCA dimension changes; values below 1 favor the signal, and the dotted vertical line marks the prespecified 64 components. Panel (**B**) reports overall alarm rates across the 60 model-condition rows at *α* = 0.01, 0.05, and 0.10.

Increasing the retained PCA dimension raised detectability but did not produce a stable Brier-harm transfer advantage (Figure 6). PCA alarm rates rose from 0.917 at 16 and 32 components to 0.933 at 64 and 128 components and 0.983 at 256 components. The dimension audit refitted PCA and used a dedicated RFF realization with 499 permutations at each dimension; consequently, its 64-component alarm rate (0.933) is a sensitivity rerun rather than an exact reproduction of the frozen 64-component, 999-permutation estimate (0.967). Relative to the training-family mean baseline, PCA mean-displacement MAE ratios were 0.964, 1.058, 1.038, 1.216, and 1.362 for 16, 32, 64, 128, and 256 components, respectively; corresponding PCA RFF-MMD ratios were 1.002, 1.116, 1.145, 1.169, and 1.135. The isolated value below 1 at 16 components is descriptive and was not accompanied by confirmatory superiority inference. The conclusion that broad PCA detectability is not sufficient evidence of Brier harm therefore did not depend on selecting 64 components.

At *α* = 0.01, 0.05, and 0.10, overall PCA alarm rates were 0.900, 0.967, and 0.967; the corresponding five-logit rates were 0.417, 0.517, and 0.533. At *α* = 0.05, PCA detected 90% of low-severity and all medium/high conditions, whereas logit monitoring detected 45%, 55%, and 55%. This confirms that threshold and severity affect power without eliminating the gap between monitored spaces.

Signal-stage computation for a 200-case window was small after shared CNN inference (Table 8). Median incremental latency ranged from 0.019 ms for logit mean displacement to 6.075 ms for the calibrated high-error selector. Persistent fitted state ranged from 0.078 KiB to 651 KiB, and approximate working arrays ranged from 7.81 KiB to 300 KiB. These measurements characterize the current implementation and workstation; they exclude image decoding and neural-network inference and are not end-to-end deployment benchmarks.

### 4.9. Exploratory Architecture Sensitivity

Both additional heads passed the frozen exploratory quality gate. Clean-monitor macro AUC was 0.765 for ConvNeXtV2-Atto and 0.772 for MobileViT-XS; all five label AUCs exceeded 0.65 for both models (Table 9). Representation RFF-MMD alarmed on 28/30 and 29/30 image conditions, whereas logit RFF-MMD alarmed on 20/30 and 16/30. The validation-reference versus clean-test diagnostic remained close to nominal: representation alarms occurred in 1/30 and 0/30 episodes, and logit alarms in 2/30 for each model.

The exact controls reproduced the construct separation in both architectures. All six task-null interventions triggered the representation RFF-MMD alarm (*p* = 0.001–0.032), none triggered the logit alarm (*p* = 0.116–0.896), the maximum absolute logit change was 9.77 × 10^−15^, and paired Brier harm was no greater than 1.39 × 10^−17^. Conversely, the 0.50 and 1.00 task-aligned offsets triggered all four corresponding logit alarms (*p* = 0.001) and increased Brier score, while representation RFF-MMD did not alarm for any of the six task-aligned controls. This reversal reinforces that the two monitors answer different questions rather than forming a universal sensitivity ordering. Because both exploratory heads achieved macro AUC above 0.75 and passed every per-label AUC floor, their replication of the exact task-null result reduces the concern that this construct separation arose solely from the sub-threshold DenseNet checkpoints; it does not make the exploratory arm a confirmatory one.

In strict bidirectional held-architecture/held-family transfer, probability mean displacement had the lowest Brier-harm MAE ratio relative to the training-family mean baseline (0.404; 18/20 folds improved), followed by probability RFF-MMD (0.468; 19/20), logit mean displacement (0.505; 16/20), PCA-representation mean displacement (0.540; 16/20), logit RFF-MMD (0.546; 19/20), and PCA-representation RFF-MMD (0.588; 18/20). The calibrated selector and mean error proxy had ratios of 0.757 and 0.849; raw-pixel mean displacement was the only signal above the baseline, at 1.116. Severity rank was also above the baseline (1.008). These exploratory point results extend the qualitative construct finding beyond DenseNet-121, but they do not convert the primary cross-checkpoint analysis into prospective architecture-general evidence.

## 5. Discussion

### 5.1. Principal Finding

The central result is not a detector ranking. It is an empirical demonstration that detectability, frozen-head relevance, and performance deterioration can be separated. The whole-model-null control is decisive because there is no ambiguity about its effect on the released classifier layer: the representation changes, yet all 18 output slots are invariant to float64 tolerance. A PCA-representation alarm is therefore correct about distribution change but uninformative about harm mediated through those released outputs. Conversely, the task-aligned control tests whether head-aware monitoring responds when the five analyzed outputs are deliberately changed.

The primary held-family analysis provides a second, independent result. Logit mean, probability mean, and probability RFF-MMD each outperformed both prespecified baselines after correction across 18 comparisons for Brier harm. PCA-representation and raw-pixel summaries did not. This supports a limited claim that head-aware displacement carried cross-checkpoint information about paired Brier deterioration in this stress-test panel. The secondary audit shows why that conclusion must remain outcome-specific: PCA-representation RFF-MMD had the best descriptive transfer ratios for AUC and locked-F1 loss.

The exploratory arm is best interpreted as a controlled architecture replication. Holding the cohort, role indices, label panel, linear-head training, perturbations, and estimands fixed isolates encoder family as the principal changed design factor. Both above-0.75-macro-AUC heads repeated the exact task-null separation to numerical precision, and output-facing signals again gave the lowest Brier-harm transfer ratios. This materially weakens a single-architecture or low-quality-model explanation for the qualitative construct separation. However, PCA-representation signals also performed below the training-mean baseline in this smaller-harm arm; the result supports the distinction between detectability and harm, not a fixed universal signal ranking or clinical-system generalization.

This distinction matters operationally. An embedding alarm may justify investigation into acquisition, preprocessing, or data provenance even when immediate predictions are stable. A harm-targeted alarm supports a different action, such as human review, recalibration, or rollback. Systems should therefore report what an alarm measures rather than collapse every alert into “model degradation.”

### 5.2. PCA-Representation Alarms Did Not Predict Primary Brier Harm

PCA-representation monitoring detected 97% of image conditions and every whole-model-null control, yet neither PCA-representation mean displacement nor PCA-representation RFF-MMD outperformed the training-family mean Brier-harm baseline after Holm correction. Their bootstrap median differences from that baseline were 0.00018 and 0.00134, respectively, with adjusted *p* = 1.0000 and 1.0000. The exact-head audit supplies a two-sided mechanism result. At the median condition, 12.6–13.5% of paired feature-change energy lay in the selected five-output task row space—25.8–27.7× the isotropic expectation—yet the remaining 86.5–87.4% made no linear contribution to those logits. This does not prove that null-space energy caused the transfer result—the alarm uses PCA/RFF geometry, and condition severity can move both components and harm—but it shows why representation signals can correlate with severity while still being dominated by task-null change. In this panel, broad detectability was therefore not cross-checkpoint evidence of Brier harm. This result revises the earlier conference recommendation: representation monitoring remains useful for distribution change, but it cannot be promoted to an outcome-unspecified degradation claim without task-aligned or delayed-label validation.

### 5.3. Harm Prediction Is Outcome-Dependent

A monitor can rank conditions similarly yet fail to calibrate the magnitude of held-family harm. This distinction explains why the probability mean remained positively rank-associated with every outcome, while its transfer ratios reversed between proper-score/calibration outcomes and discrimination/thresholded outcomes. The probability–Brier relationship also benefits from shared functional inputs, whereas AUC depends on ordering and locked F1 on a decision threshold. Outcome-conditional recommendations are therefore more defensible than a universal detector ranking: use probability-facing summaries when the monitored target is probabilistic accuracy or calibration, and retain broad representation summaries as candidates when discrimination or thresholded utility is the target. These are hypotheses supported by this stress-test panel, not deployment guarantees.

Together, the audits identify three analyst-relative constructs: detectability is relative to the retained monitored subspace, task relevance is relative to the outputs actually served, and harm is relative to the chosen performance outcome. The whole-model-null extension removes output-selection ambiguity for the released classifier layer, but these three scopes must still be declared in any operational use.

The post hoc component signals further separate prediction from mechanism. For Brier harm, exact-head task-row and task-null mean-displacement ratios were similar rather than showing an inert-component advantage: 0.822 versus 0.845 under the deployment-reference definition, and 0.842 versus 0.847 under the paired-clean definition. For AUC loss, the null-component ratios were lower (0.662 and 0.656) than the task-row ratios (0.723 and 0.725). The 25.8–27.7× task-row enrichment shows that the two component magnitudes co-vary under these ordered corruptions rather than behaving as independent mechanisms. A causally inert component can therefore remain predictively associated as a severity proxy. These four signals receive no superiority inference and are not proposed as operational monitors.

### 5.4. Relationship to Prior Work

The study does not replace sequential harmful-shift monitoring. Amoukou et al. provide a stronger streaming framework and false-alarm theory for a calibrated high-error selector [9]; WATCH provides adaptive martingale monitoring and diagnostic categories [11]. Radiology-specific work has applied statistical process control to radiological data streams [27] and ensembled monitoring to real-time assessment of an intracranial-hemorrhage detector [28]. Our contribution is an orthogonal validation instrument against which those or other monitors can be tested. Similarly, Roschewitz et al. identify types of medical-image shift from representations and outputs [10]. We show why those spaces should be interpreted as different views of the model rather than assumed to encode harm.

The findings also revise the earlier thesis-derived detector comparison [16]. The conference study asked which detector raised reliable alarms across its scenarios and backbones. The new evidence shows that cross-architecture detection reliability is insufficient for a harm claim. MMD remains useful for detecting distribution change, but the action following an alarm must depend on task alignment and delayed performance evidence.

### 5.5. Practical Monitoring Architecture

A defensible deployment stack should retain at least three layers. First, an image or representation monitor detects acquisition and population change. Second, a head-aware or calibrated error proxy estimates whether the frozen task is likely to be affected. Third, delayed labels audit realized discrimination and calibration. The layers should not vote as interchangeable detectors: they answer different questions. In practice, a PCA alarm without output change should trigger investigation of acquisition settings, preprocessing, data provenance, device mix, or routing—not an automatic claim of clinical degradation or rollback. Escalation to human review, recalibration, or rollback requires corroborating head-aware evidence, delayed labels, or a prespecified operational rule. Exact null testing against the complete deployed output set can be added during pre-deployment verification to document how much representation change the released head can ignore. The released checkpoints also illustrate a practical audit requirement: seven advertised pathology slots—Infiltration, Emphysema, Fibrosis, Pleural Thickening, Nodule, Mass, Hernia—have denormal-scale classifier rows and therefore produce channels that are constant to operating precision and cannot contribute useful information to an output-space monitor. Output monitors should remove or explicitly account for structurally inactive channels rather than assume every advertised slot is live. This layered interpretation is consistent with production-monitoring architectures and current health-care monitoring guidance that link heterogeneous signals to declared response pathways rather than relying on a single *p*-value [29,41,42].

Simple mean displacements remained necessary baselines. In this implementation, linear mean displacement and RFF-MMD are the same mean-difference operation under different feature maps. The nonlinear RFF map can encode distributional differences beyond the linear PCA coordinates, but it added little condition-ordering information in this panel; its remaining value lies in permutation-calibrated testing or sensitivity to changes exposed only by the nonlinear map. The directional audit also defines the scope of that sensitivity: the leading-PC task-null control was not uniquely favorable among retained or random null directions, but any change exactly orthogonal to the retained 64-PC span is invisible to this arm by construction. The learned-error selector may still be useful when paired with a demonstrably predictive error model, but the low R^2^ values here prevent a strong comparative conclusion.

### 5.6. Limitations

First, neither public source model crossed the frozen 0.75 external macro-AUC gate. A weaker baseline can compress or distort the dynamic range of calibration, error, and shift-induced harm and may change signal–outcome relationships; a stronger classifier could therefore produce different rankings. The exact algebraic invariance control does not depend on baseline AUC, but all empirical harm-transfer results do. The two exploratory heads achieved macro AUCs above 0.75 and reproduced the exact construct separation, reducing—but not eliminating—the possibility that this qualitative result is an artifact of the weaker DenseNet checkpoints. The primary harm-transfer ranking remains limited to monitoring constructs, and architecture-specific or clinical conclusions require stronger, independently validated source models.

Second, validation-to-test patient disjointness is supported by published ChestMNIST split provenance rather than reverified locally, because the packaged archive contains no patient identifiers. Calibration and reference are image-disjoint subsets of the validation split, but patient-level separation between those two source-side roles is unknown; repeated patients could make those source-side estimates more dependent. Third, the 30 conditions are algorithmic stressors. Their low/medium/high labels are parameter-order categories, not clinical severity grades. They increase condition-level diversity but do not reproduce natural site, device, temporal, prevalence, workflow, or population shifts; “cross-checkpoint” is therefore more accurate than an unrestricted claim of deployment transferability. Fourth, the confirmatory held-source-domain transfer remains limited to two DenseNet-121 checkpoints. The exploratory arm is a controlled architecture replication: holding the cohort, head-training procedure, perturbation panel, and estimands fixed reduces confounding and shows that the exact construct separation persists in two above-0.75-macro-AUC encoder families. The same controls that strengthen architecture attribution also limit external generalization: the arm does not test different clinical training datasets, independently developed clinical heads, natural deployment streams, or prospective model selection, and it was specified only after the primary analysis. Fifth, the common-offset task-aligned control is rank-preserving: it cannot change AUC and therefore does not provide a controlled discrimination-harm intervention. Discrimination is exercised only by the image-condition panel. Sixth, PCA was fitted on the 2000-image reference pool from which 200-case reference test windows were sampled, creating residual fit–test dependence. The empirical null checks were reassuring but do not replace a disjoint transform-fitting role. The PCA-dimension sensitivity audit shows that changing the retained dimension alters power but does not create a stable Brier-harm advantage; it does not remove the dependence. Seventh, nullity is relative to a specified output set. The post hoc whole-model control covers every output of the current released linear classifier layer, but it would not guarantee invariance after a head update, an added output, or use of the representation by another downstream consumer. Eighth, Brier harm depends on the fixed calibration procedure and five-label panel. Pneumonia and pneumothorax were active common labels outside that prespecified panel, and a different panel could change metric-dependent rankings. The whole-model-null audit resolves only the invariance scope. The secondary transfer, exact-head mechanism, calibration-removal, dimension, threshold, resource, and architecture-sensitivity audits are post hoc or exploratory and descriptive; they require prospective confirmation and do not establish clinical utility.

Ninth, the Amoukou-style comparator uses a weak error regressor and reproduces its calibrated-selector principle but not the complete sequential confidence-sequence algorithm. Its result cannot establish inferiority of that method. The finite condition design evaluates batch harm prediction, not detection delay. Finally, the condition panel contains three ordered severities per family; cluster bootstrap respects family structure but cannot identify how the results generalize to all possible natural shifts.

Live radiology deployments also fail through routing, utilization, infrastructure, and human-workflow pathways that are absent from a transformed-image benchmark; recent monitoring experience across 17 deployed algorithms illustrates the importance of these operational signals [43].

Related systems work further separates model-output monitoring from the operational layers required for safe deployment: remote update and rollback, resilient e-health service architecture, and device-level clinical monitoring address complementary failure and recovery pathways [44,45,46].

### 5.7. Future Validation

The next study should use clinically stronger source classifiers, patient-level identifiers, at least one untouched temporal or institutional stream, and prospective delayed labels. The present exploratory ConvNeXtV2/MobileViT comparison should be repeated in a prospectively specified panel whose architectures, training domains, heads, and quality gates are fixed before any shift outcomes are inspected. A disjoint validation slice should fit PCA and kernel parameters before an independent reference slice supplies alarm windows. The study should retain a null control defined against the complete deployed output set and prospectively add a label-independent, per-image, rank-changing intervention to exercise discrimination without selecting its form or magnitude from observed outcomes. Any learned-error comparison should require a prespecified out-of-sample quality floor before its monitoring result is interpreted.

## 6. Conclusions

A distribution-shift alarm is evidence that a monitored representation changed; it is not automatically evidence that a classifier was harmed. A whole-model-null intervention made that distinction observable while preserving every output of the released classifier layer. The post hoc selected-head decomposition supplied both sides of the mechanism: task-row energy was 25.8–27.7× above the isotropic expectation, while about 86–87% of paired per-image energy remained task-null at the median condition. For primary Brier harm, PCA-representation monitoring detected nearly every image condition and every whole-model-null control, but neither PCA-representation signal outperformed the training-mean baseline after multiplicity correction; only output-facing signals were superior to both prespecified baselines. PCA-dimension sensitivity did not create a stable PCA advantage. In descriptive secondary-outcome transfer, however, PCA-representation RFF-MMD had the lowest ratios for macro-AUC and locked-F1 loss. There was no outcome-invariant winner. The exploratory ConvNeXtV2-Atto/MobileViT-XS arm reproduced exact task-null separation in two above-0.75-macro-AUC heads and placed probability mean first in held-architecture/held-family Brier-harm transfer, but remains a controlled, same-cohort exploratory replication rather than prospective clinical-system validation. The resulting recommendation is layered and outcome-aware: use representation monitoring for distribution change, validated head-aware proxies for a specified performance target, and delayed labels for realized performance. The study supplies a reproducible validation framework across two clinical checkpoints and two additional architecture families, while deliberately stopping short of prospective architecture-generalization or clinical-safety claims.

## Figures and Tables

**Table 1:** External-domain quality of the two frozen source models on the full ChestMNIST test split.

Source Model	Test Cases	Macro AUC	Macro F1 at Distributed Source Thresholds	Mean Brier
CheXpert-trained	22,433	0.733	0.176	0.193
MIMIC-CXR-trained	22,433	0.714	0.160	0.112

**Table 2:** Prespecified image-shift generation, low/medium/high parameter levels, and stress-test rationale. Intensity-scale quantities use images normalized to [0, 1]. These algorithmic conditions span distinct acquisition-like perturbation axes; they are not validated simulations of a particular hospital, device, or population shift.

Family	Low	Medium	High	Rationale
Brightness	+0.03	+0.06	+0.12	Global exposure/level displacement
Contrast	+0.15	+0.30	+0.50	Contrast factor 1 + s about the reference mean
Gamma	1.15	1.35	1.60	Nonlinear intensity-response change
Gaussian noise	SD 0.02	SD 0.05	SD 0.10	Increasing additive sensor-like noise
Blur	blend 0.25	blend 0.50	blend 0.80	Blend with a 3 × 3 average-filtered image
Rotation	3 degrees	7 degrees	12 degrees	Positioning/orientation perturbation
Resolution	retain 75%	retain 50%	retain 35%	Downsample then restore to 224 × 224
Central occlusion	5% area	10% area	20% area	Missing central image content
Vignette	attenuation 0.10	attenuation 0.25	attenuation 0.40	Radial sensitivity/bias-field change
Mixed	brightness 0.02; blur 0.20; noise 0.01	brightness 0.05; blur 0.40; noise 0.03	brightness 0.09; blur 0.65; noise 0.06	Fixed combination of mild-to-strong acquisition-like effects

**Table 3:** Cross-validated error-proxy quality and calibrated high-error-selector operating characteristics.

Model	Error-Proxy Cross-Validated R^2^	Error-Proxy MAE	Selector Power	Selector FDP	Selected Predicted-Error Quantile
CheXpert-trained	0.058	0.0622	0.801	0.199	0.50
MIMIC-CXR-trained	0.081	0.0609	0.672	0.160	0.60

**Table 4:** Calibrated clean-monitor performance of the two frozen source models.

Model	Macro AUC	Macro F1	Mean Brier	Mean ECE
CheXpert-trained	0.732	0.211	0.056	0.012
MIMIC-CXR-trained	0.715	0.209	0.056	0.011

**Table 5:** Exact-head decomposition of cached paired clean-to-condition feature changes. The isotropic expectation is the row-space rank divided by representation dimension. Per-image fractions are ratios of summed squared energy within each condition, and are summarized by the median across 30 conditions.

Source Model	Displacement Summary	Median Task-Row Energy Fraction	Enrichment over 5/1024 Isotropic Expectation
CheXpert-trained	Paired mean displacement	0.078	16.0×
MIMIC-CXR-trained	Paired mean displacement	0.117	24.1×
CheXpert-trained	Paired per-image energy	0.135	27.7×
MIMIC-CXR-trained	Paired per-image energy	0.126	25.8×

**Table 6:** Held-source-domain and held-family prediction of primary paired Brier harm. Negative bootstrap differences favor the signal over the training-family mean baseline.

Unlabeled Signal	Point MAE	Folds Better than Baseline	Bootstrap MAE Difference from Training Mean, Median [95% Interval]	Maximum Holm-Adjusted *p* Across Two Baselines
PCA-representation mean displacement	0.01534	17/20	0.00018 [−0.00485, 0.00121]	1.0000
PCA-representation RFF-MMD	0.01643	14/20	0.00134 [−0.00329, 0.00273]	1.0000
Logit mean displacement	0.01041	16/20	−0.00453 [−0.00896, −0.00082]	≤0.0072
Logit RFF-MMD	0.01306	13/20	−0.00226 [−0.00499, −0.00010]	0.3079
Probability mean displacement	0.00679	18/20	−0.00798 [−0.01411, −0.00228]	≤0.0072
Probability RFF-MMD	0.01257	14/20	−0.00273 [−0.00573, −0.00050]	≤0.0072
Raw-pixel projection mean displacement	0.01480	13/20	0.00050 [−0.00005, 0.00283]	1.0000
Mean learned-error proxy	0.01766	13/20	0.00265 [−0.00244, 0.00639]	1.0000
Calibrated high-error selector	0.01546	11/20	0.00026 [−0.00259, 0.00185]	1.0000

**Table 7:** Descriptive held-source-domain/held-family MAE ratios relative to the outcome-specific training-family mean-harm baseline. Values below 1 indicate lower error. The five outcome analyses use identical folds and ridge mappings; only the Brier analysis was prespecified for confirmatory transfer inference.

Unlabeled Signal	Brier	Log Loss	ECE	Macro-AUC Loss	Locked-F1 Loss
PCA-representation mean displacement	1.038	0.982	0.936	0.603	0.679
PCA-representation RFF-MMD	1.112	1.054	1.030	0.570	0.609
Logit mean displacement	0.705	0.621	0.629	0.931	1.094
Logit RFF-MMD	0.884	0.820	0.815	0.586	0.768
Probability mean displacement	0.459	0.329	0.301	1.630	1.740
Probability RFF-MMD	0.850	0.782	0.775	0.585	0.748
Raw-pixel projection mean displacement	1.002	1.018	1.017	0.917	0.934
Mean learned-error proxy	1.195	1.154	1.150	0.760	0.777
Calibrated high-error selector	1.046	1.011	1.010	0.697	0.797

**Table 8:** Incremental signal-stage resource profile for one 200-case monitoring window, averaged across the two checkpoints. State is fitted persistent data; working memory approximates arrays materialized for one window.

Signal	Median Latency (ms)	State (KiB)	Working Memory (KiB)
Logit mean displacement	0.019	0.078	7.81
Raw-pixel projection mean displacement	0.224	1.50	100.0
PCA mean displacement	0.766	520.5	100.0
Logit RFF-MMD	0.968	13.08	207.8
Probability mean displacement	0.949	0.195	7.81
Probability RFF-MMD	2.043	13.20	207.8
PCA RFF-MMD	2.195	651.0	300.0
Mean learned-error proxy	5.793	474.0	39.1
Calibrated high-error selector	6.075	474.0	39.1

**Table 9:** Exploratory architecture-sensitivity summary. The models use frozen ImageNet encoders with five ChestMNIST-trained linear heads; the arm is descriptive and separate from the confirmatory two-checkpoint estimand.

Model	Representation Dimension	Clean Macro AUC	Clean Macro F1	Representation Alarms	Logit Alarms	Median Paired Brier Harm	Maximum Paired Brier Harm
ConvNeXtV2-Atto	320	0.765	0.222	28/30	20/30	0.00060	0.01262
MobileViT-XS	384	0.772	0.235	29/30	16/30	0.00039	0.00827

## Data Availability

The data analyzed in this study are publicly available: ChestMNIST (MedMNIST v2) at https://medmnist.com (accessed on 4 October 2026) and the TorchXRayVision model weights at https://github.com/mlmed/torchxrayvision (accessed on 4 October 2026). The source code, condition-level results, and reproducibility archive are provided in the Supplementary Materials. Further inquiries can be directed to the corresponding author.

## Supplementary Materials

The following supporting information can be downloaded at: https://www.mdpi.com/article/10.3390/jimaging12100492/s1, Document S1: Supplementary Methods and Results (Sections S1–S14), including complete shift settings, output-slot scope, sensitivity analyses, and resource profiles; Archive S1: reproducibility archive containing the frozen protocol, model and cache manifests, condition-level CSV, exact-control audit, analysis JSON, source code, environment record, and figure-generation scripts.
