# Supplementary material for: Detectable, Task-Relevant, or Harmful? Construct-Valid Evaluation of Unlabeled Distribution-Shift Monitoring in Chest Radiography
Source: J Imaging. 2026 Oct 7;12(10):492. doi: 10.3390/jimaging12100492 (PMC13705775; doi:10.3390/jimaging12100492)
Supplement: Supplementary file 1 [file jimaging-12-00492-s001.zip › jimaging-4571871-supplementary.pdf]

Supplementary Methods and Results

Companion to *Detectable, Task-Relevant, or Harmful? Construct-Valid Evaluation of Unlabelled Distribution-Shift Monitoring in Chest Radiography*.

Authors: Dan-Gabriel Badea; Răzvan-Andrei Rotaru; Răzvan-Victor Rughiniș; Flavia Zaim-Oprea; Dinu Țurcanu; Daniel Rosner.

S1. Frozen design and provenance

The protocol was frozen on 24 August 2026 before extraction of the new shifted-image outcomes. The analysis used seed 20260824, two public TorchXRayVision DenseNet-121 models, and ChestMNIST v2 at 224 pixels. Role sizes were 4000 calibration, 2000 reference, and 2000 monitor images. Calibration/reference roles came from the official validation split; monitoring came from the official test split.

Environment: Python 3.12.10 (tags/v3.12.10:0cc8128, Apr 8 2025, 12:21:36) [MSC v.1943 64 bit (AMD64)]; PyTorch 2.12.1+cpu; NumPy 1.26.4; TorchXRayVision 1.5.4; MedMNIST 3.0.2.

The 64-component whitened PCA basis was estimated from all 2000 reference-role images. Each alarm episode then sampled 200 reference cases from that fitted pool, so the transform and reference test window are not fully independent. Both within-reference and across-split clean diagnostics below empirically evaluate this complete fit–test pipeline.

The large feature-cache directory is not included in the submission package because its contents are derived and regenerable from the public inputs and supplied scripts. The role-index/label manifest SHA-256 is d000cb2ba609eb05a17ecf737c0db0c47e6d021b1b6b32e281aaf89ce3e46ac5.

A runtime-change equivalence audit covered 11 pre-existing condition caches; all passed exact checks and 6 were migrated. The audit records full-file hashes, full 2000-case pixel-precursor equality, and fixed-sample equality for both models.

S2. Complete external-domain model audit

Source model	Label	Positives	Prevalence	AUC	F1 at distributed threshold	Brier	Distributed threshold
CheXpert-trained	atelectasis	2420	0.1079	0.683	0.263	0.236	0.1989
CheXpert-trained	cardiomegaly	582	0.0259	0.681	0.076	0.201	0.1971
CheXpert-trained	effusion	2754	0.1228	0.765	0.350	0.211	0.2721
CheXpert-trained	consolidation	957	0.0427	0.751	0.138	0.123	0.0571
CheXpert-trained	edema	413	0.0184	0.784	0.055	0.194	0.1435
MIMIC-CXR-trained	atelectasis	2420	0.1079	0.682	0.244	0.155	0.0912
MIMIC-CXR-trained	cardiomegaly	582	0.0259	0.635	0.064	0.143	0.0501
MIMIC-CXR-trained	effusion	2754	0.1228	0.775	0.321	0.141	0.0800
MIMIC-CXR-trained	consolidation	957	0.0427	0.702	0.115	0.046	0.0106

Source model	Label	Positives	Prevalence	AUC	F1 at distributed threshold	Brier	Distributed threshold
MIMIC-CXR-trained	edema	413	0.0184	0.775	0.054	0.074	0.0037

Neither model met the frozen macro-AUC gate of 0.75. The protocol specified no joint-failure contingency; Amendment 4 records the post-audit decision to retain both prespecified models as sub-threshold instruments and restrict interpretation to construct validation.

S3. Complete image-condition results

Each row is one source-domain model instance and shift-condition mean based on the same 2000 monitor images. The 60 rows are paired observations from 30 condition clusters, not 60 independent inferential units. The family-cluster bootstrap retains both model observations.

Model	Condition	Paired Brier harm	Delta macro AUC	Delta macro F1	Delta ECE	PCA RFF-MMD	Logit RFF-MMD	PCA p	Logit p	Error-selector change
chexpert	brightness_0.03	0.00012	-0.00066	-0.00011	0.00007	0.003620	0.001079	0.001	0.416	0.01000
chexpert	brightness_0.06	0.00034	-0.00592	0.00213	0.00153	0.013908	0.001782	0.001	0.319	-0.00050
chexpert	brightness_0.12	0.00124	-0.02524	-0.01172	0.00286	0.063739	0.004437	0.001	0.125	0.03250
chexpert	contrast_0.15	-0.00031	0.00722	0.00646	0.00089	0.010460	0.000471	0.001	0.216	-0.00650
chexpert	contrast_0.3	-0.00054	0.01173	0.00855	0.00459	0.041832	0.002388	0.001	0.061	0.00000
chexpert	contrast_0.5	-0.00068	0.01295	0.01518	0.00452	0.104487	0.009026	0.001	0.110	0.03950
chexpert	gamma_1.15	-0.00015	-0.00385	-0.00107	-0.00089	0.004764	0.002426	0.002	0.210	-0.02550
chexpert	gamma_1.35	-0.00015	-0.00958	-0.00796	-0.00083	0.020240	0.011303	0.001	0.004	-0.03250
chexpert	gamma_1.6	0.00023	-0.01847	-0.03165	0.00170	0.050280	0.032249	0.001	0.001	-0.02800
chexpert	noise_0.02	-0.00023	0.00069	0.00243	0.00067	0.105567	0.021165	0.001	0.017	0.12550
chexpert	noise_0.05	0.00350	-0.07241	-0.07954	0.01888	0.662718	0.343643	0.001	0.001	0.46350
chexpert	noise_0.1	0.00496	-0.13608	-0.11979	0.01728	0.796202	0.636746	0.001	0.001	0.44700
chexpert	blur_0.25	0.00006	-0.00119	0.00061	-0.00049	0.001066	0.001312	0.368	0.261	0.00250
chexpert	blur_0.5	0.00011	-0.00267	-0.00100	-0.00044	0.002199	0.001851	0.018	0.433	0.00650

Model	Condition	Paired Brier harm	Delta macro AUC	Delta macro F1	Delta ECE	PCA RFF-MMD	Logit RFF-MMD	PCA p	Logit p	Error-selector change
chexpert	blur_0.8	0.00018	-0.00462	-0.00067	-0.00088	0.004065	0.002845	0.002	0.317	0.02150
chexpert	rotation_3	0.00006	-0.00252	-0.00035	-0.00080	0.013766	0.001685	0.001	0.553	-0.01300
chexpert	rotation_7	0.00008	-0.00293	-0.00238	-0.00059	0.032654	0.003843	0.001	0.129	-0.01500
chexpert	rotation_12	0.00012	-0.00341	0.00025	-0.00078	0.053698	0.006063	0.001	0.162	-0.00750
chexpert	resolution_0.75	0.00019	-0.00512	-0.00260	-0.00130	0.003646	0.003711	0.024	0.113	0.01400
chexpert	resolution_0.5	0.00029	-0.00839	-0.00699	-0.00116	0.007290	0.006197	0.001	0.021	0.03200
chexpert	resolution_0.35	0.00061	-0.01559	-0.01052	0.00139	0.016260	0.026921	0.001	0.001	0.10100
chexpert	occlusion_0.05	0.00108	-0.03757	-0.02275	-0.00149	0.071879	0.066787	0.001	0.001	0.05650
chexpert	occlusion_0.1	0.00179	-0.04936	-0.04841	-0.00088	0.094077	0.116386	0.001	0.001	0.15100
chexpert	occlusion_0.2	0.00258	-0.09777	-0.08171	-0.00494	0.284619	0.192648	0.001	0.001	0.16050
chexpert	vignette_0.1	0.00002	-0.00245	-0.00210	-0.00082	0.002077	0.002620	0.007	0.020	0.00600
chexpert	vignette_0.25	0.00007	-0.00700	-0.00272	-0.00082	0.006775	0.008281	0.001	0.001	0.01750
chexpert	vignette_0.4	0.00016	-0.01283	-0.01284	-0.00062	0.013990	0.019608	0.001	0.002	0.02950
chexpert	mixed_1	-0.00009	0.00121	0.00110	0.00097	0.021566	0.002752	0.001	0.432	0.03850
chexpert	mixed_2	0.00131	-0.01739	-0.02411	0.00962	0.313615	0.117675	0.001	0.001	0.31700
chexpert	mixed_3	0.00670	-0.09580	-0.08516	0.02778	0.732623	0.454343	0.001	0.001	0.47550
mimic_ch	brightness_0.03	0.00016	-0.01251	-0.00677	-0.00212	0.003599	0.002606	0.014	0.378	-0.01600
mimic_ch	brightness_0.06	0.00056	-0.03210	-0.02647	-0.00157	0.014938	0.004509	0.001	0.145	-0.02350

Model	Condition	Paired Brier harm	Delta macro AUC	Delta macro F1	Delta ECE	PCA RFF-MMD	Logit RFF-MMD	PCA p	Logit p	Error-selector change
mimic_ch	brightness_0.12	0.00242	-0.07422	-0.05969	0.00664	0.111154	0.009129	0.001	0.041	-0.06600
mimic_ch	contrast_0.15	-0.00040	0.01032	0.00726	-0.00072	0.019263	0.004680	0.001	0.526	-0.02700
mimic_ch	contrast_0.3	0.00026	0.00410	0.00199	0.00272	0.111022	0.019674	0.001	0.002	-0.07800
mimic_ch	contrast_0.5	0.00177	-0.01539	-0.01105	0.01154	0.272663	0.047265	0.001	0.001	-0.13100
mimic_ch	gamma_1.15	0.00013	0.00258	-0.00397	0.00051	0.006481	0.003664	0.001	0.002	-0.03000
mimic_ch	gamma_1.35	0.00078	-0.01108	-0.02106	0.00456	0.033271	0.026018	0.001	0.001	-0.01450
mimic_ch	gamma_1.6	0.00122	-0.02837	-0.04541	0.00709	0.085916	0.066338	0.001	0.001	0.00100
mimic_ch	noise_0.02	0.00298	0.01258	-0.01580	0.02554	0.164542	0.052671	0.001	0.001	0.23150
mimic_ch	noise_0.05	0.10634	-0.05020	-0.08071	0.21824	0.631833	0.568820	0.001	0.001	0.56950
mimic_ch	noise_0.1	0.16732	-0.14797	-0.08197	0.26340	0.710001	0.865427	0.001	0.001	0.58700
mimic_ch	blur_0.25	0.00012	-0.00123	-0.00070	0.00005	0.001210	0.000396	0.326	0.709	-0.00900
mimic_ch	blur_0.5	0.00023	-0.00269	-0.00190	-0.00053	0.003081	0.000578	0.001	0.806	-0.00950
mimic_ch	blur_0.8	0.00034	-0.00436	-0.00758	-0.00091	0.006543	0.001451	0.001	0.746	-0.02100
mimic_ch	rotation_3	0.00016	-0.00236	-0.00822	0.00049	0.008209	0.001571	0.001	0.399	-0.04450
mimic_ch	rotation_7	0.00019	-0.00083	-0.00364	0.00123	0.014108	0.002607	0.001	0.397	-0.04100
mimic_ch	rotation_12	0.00023	-0.00144	-0.00376	0.00118	0.019683	0.003958	0.001	0.218	-0.06700
mimic_ch	resolution_0.75	0.00017	-0.00144	-0.00506	0.00124	0.005252	0.001618	0.001	0.249	0.00450
mimic_ch	resolution_0.5	0.00048	-0.00727	-0.00740	-0.00004	0.013829	0.003337	0.001	0.361	0.00050
mimic_ch	resolution_0.35	0.00100	-0.01512	-0.01914	0.00161	0.041703	0.012519	0.001	0.006	0.07550
mimic_ch	occlusion_0.05	0.00246	-0.04470	-0.06735	0.00406	0.060620	0.016803	0.001	0.012	-0.05000

Model	Condition	Paired Brier harm	Delta macro AUC	Delta macro F1	Delta ECE	PCA RFF-MMD	Logit RFF-MMD	PCA p	Logit p	Error-selector change
mimic_ch	occlusion_0.1	0.00344	-0.08125	-0.12567	0.00321	0.112076	0.039911	0.001	0.001	-0.02600
mimic_ch	occlusion_0.2	0.00351	-0.09391	-0.10566	0.00200	0.270739	0.124837	0.001	0.001	0.21950
mimic_ch	vignette_0.1	0.00016	-0.00346	-0.00374	0.00046	0.001478	0.002244	0.017	0.643	0.00600
mimic_ch	vignette_0.25	0.00057	-0.01128	-0.01337	0.00016	0.005383	0.005883	0.002	0.595	0.00400
mimic_ch	vignette_0.4	0.00119	-0.02381	-0.02323	0.00136	0.013007	0.008690	0.001	0.282	0.02700
mimic_ch	mixed_1	0.00016	-0.00315	-0.00420	0.00177	0.020795	0.011009	0.001	0.022	0.06900
mimic_ch	mixed_2	0.01543	-0.04897	-0.04910	0.05729	0.382826	0.124957	0.001	0.001	0.32500
mimic_ch	mixed_3	0.14340	-0.14037	-0.08664	0.25923	0.675575	0.743567	0.001	0.001	0.58500

S4. Exact representation-control audit

For selected-output-null rows, "audit error" is the maximum absolute selected-logit change. For task-aligned rows, it is the maximum absolute deviation from the requested common logit offset. All calculations are float64. Control MMD values and permutation p-values compare altered monitor representations with the corresponding clean-monitor pool, isolating the intervention from the validation-to-test sampling boundary. These are the frozen confirmatory controls; the stricter post hoc whole-model-null extension is reported in Section S10.

Model	Control	Audit error	Paired Brier harm	Delta macro AUC	Delta locked F1	PCA RFF-MMD	Logit RFF-MMD	PCA p	Logit p
chexpert	tasknull_0.25	6.217e-15	-0.00000000	0.0000000	0.0000000	0.0509415	0.0000000	0.001	0.121
chexpert	tasknull_0.5	6.217e-15	-0.00000000	0.0000000	0.0000000	0.2041259	0.0000000	0.001	0.703
chexpert	tasknull_1	8.882e-15	-0.00000000	0.0000000	0.0000000	0.6455455	0.0000000	0.001	0.676
chexpert	taskaligned_0.25	5.329e-15	0.00007940	0.0000000	-0.0012030	0.0028342	0.0021113	0.008	0.838
chexpert	taskaligned_0.5	6.217e-15	0.00031191	0.0000000	-0.0001396	0.0115207	0.0084636	0.001	0.001

Model	Control	Audit error	Paired Brier harm	Delta macro AUC	Delta locked F1	PCA RFF-MMD	Logit RFF-MMD	PCA p	Logit p
chexpert	taskaligned_1	5.329e-15	0.00134779	0.0000000	-0.0083237	0.0475432	0.0337996	0.001	0.001
mimic_ch	tasknull_0.25	8.882e-15	-0.00000000	0.0000000	0.0000000	0.0366225	0.0000000	0.001	0.560
mimic_ch	tasknull_0.5	7.105e-15	-0.00000000	0.0000000	0.0000000	0.1413311	0.0000000	0.001	0.402
mimic_ch	tasknull_1	7.105e-15	-0.00000000	0.0000000	0.0000000	0.4793272	0.0000000	0.001	0.172
mimic_ch	taskaligned_0.25	7.105e-15	0.00004022	0.0000000	0.0010297	0.0010480	0.0021092	0.333	0.542
mimic_ch	taskaligned_0.5	7.105e-15	0.00025778	0.0000000	-0.0035021	0.0042247	0.0084327	0.001	0.154
mimic_ch	taskaligned_1	7.105e-15	0.00134889	0.0000000	-0.0160198	0.0170117	0.0333956	0.001	0.001

The task-aligned control adds the same constant to every image's logit for a given label. It therefore preserves within-label score ordering and leaves macro AUC exactly unchanged by construction. Its six Brier changes range from 0.00004 to 0.00135. It is a controlled output-displacement/calibration probe, not a controlled discrimination-harm intervention.

The following post-analysis scale audit compares each inert control with the 30 image-condition PCA-representation RFF-MMD values for the same source model. All controls lie inside the observed image-condition range; their detection therefore does not depend on an out-of-scale representation injection.

Model	Selected-output-null magnitude	PCA RFF-MMD	Same-model image-condition range	Empirical rank among image conditions
chexpert	0.25	0.0509415	0.0010660–0.7962022	19/30 (63% empirical percentile)
chexpert	0.50	0.2041259	0.0010660–0.7962022	25/30 (83% empirical percentile)
chexpert	1.00	0.6455455	0.0010660–0.7962022	27/30 (90% empirical percentile)
mimic_ch	0.25	0.0366225	0.0012103–0.7100006	17/30 (57% empirical percentile)
mimic_ch	0.50	0.1413311	0.0012103–0.7100006	23/30 (77% empirical percentile)
mimic_ch	1.00	0.4793272	0.0012103–0.7100006	27/30 (90% empirical percentile)

S5. Complete held-source-domain and held-family folds

Signal	Training model	Test model	Held family	MAE	Training-family mean MAE
PCA mean	chexpert	mimic_ch	blur	0.00008	0.00063
PCA mean	chexpert	mimic_ch	brightness	0.00068	0.00084
PCA mean	chexpert	mimic_ch	contrast	0.00056	0.00095
PCA mean	chexpert	mimic_ch	gamma	0.00036	0.00039
PCA mean	chexpert	mimic_ch	mixed	0.05139	0.05270
PCA mean	chexpert	mimic_ch	noise	0.08660	0.09164
PCA mean	chexpert	mimic_ch	occlusion	0.00262	0.00246
PCA mean	chexpert	mimic_ch	resolution	0.00028	0.00040
PCA mean	chexpert	mimic_ch	rotation	0.00008	0.00067
PCA mean	chexpert	mimic_ch	vignette	0.00043	0.00044
PCA mean	mimic_ch	chexpert	blur	0.00628	0.01678
PCA mean	mimic_ch	chexpert	brightness	0.00354	0.01623
PCA mean	mimic_ch	chexpert	contrast	0.00499	0.01737
PCA mean	mimic_ch	chexpert	gamma	0.00274	0.01686
PCA mean	mimic_ch	chexpert	mixed	0.03929	0.00839
PCA mean	mimic_ch	chexpert	noise	0.08753	0.00393
PCA mean	mimic_ch	chexpert	occlusion	0.00896	0.01475
PCA mean	mimic_ch	chexpert	resolution	0.00414	0.01649
PCA mean	mimic_ch	chexpert	rotation	0.00169	0.01681
PCA mean	mimic_ch	chexpert	vignette	0.00459	0.01677
PCA RFF-MMD	chexpert	mimic_ch	blur	0.00022	0.00063
PCA RFF-MMD	chexpert	mimic_ch	brightness	0.00081	0.00084
PCA RFF-MMD	chexpert	mimic_ch	contrast	0.00044	0.00095
PCA RFF-MMD	chexpert	mimic_ch	gamma	0.00041	0.00039
PCA RFF-MMD	chexpert	mimic_ch	mixed	0.05089	0.05270
PCA RFF-MMD	chexpert	mimic_ch	noise	0.08812	0.09164
PCA RFF-MMD	chexpert	mimic_ch	occlusion	0.00224	0.00246
PCA RFF-MMD	chexpert	mimic_ch	resolution	0.00046	0.00040
PCA RFF-MMD	chexpert	mimic_ch	rotation	0.00008	0.00067
PCA RFF-MMD	chexpert	mimic_ch	vignette	0.00060	0.00044
PCA RFF-MMD	mimic_ch	chexpert	blur	0.00863	0.01678
PCA RFF-MMD	mimic_ch	chexpert	brightness	0.00522	0.01623
PCA RFF-MMD	mimic_ch	chexpert	contrast	0.00649	0.01737
PCA RFF-MMD	mimic_ch	chexpert	gamma	0.00401	0.01686
PCA RFF-MMD	mimic_ch	chexpert	mixed	0.05621	0.00839
PCA RFF-MMD	mimic_ch	chexpert	noise	0.06626	0.00393
PCA RFF-MMD	mimic_ch	chexpert	occlusion	0.01939	0.01475
PCA RFF-MMD	mimic_ch	chexpert	resolution	0.00722	0.01649
PCA RFF-MMD	mimic_ch	chexpert	rotation	0.00333	0.01681
PCA RFF-MMD	mimic_ch	chexpert	vignette	0.00762	0.01677
Logit mean	chexpert	mimic_ch	blur	0.00055	0.00063
Logit mean	chexpert	mimic_ch	brightness	0.00107	0.00084
Logit mean	chexpert	mimic_ch	contrast	0.00043	0.00095
Logit mean	chexpert	mimic_ch	gamma	0.00011	0.00039
Logit mean	chexpert	mimic_ch	mixed	0.04764	0.05270
Logit mean	chexpert	mimic_ch	noise	0.08025	0.09164
Logit mean	chexpert	mimic_ch	occlusion	0.00222	0.00246
Logit mean	chexpert	mimic_ch	resolution	0.00040	0.00040

Signal	Training model	Test model	Held family	MAE	Training-family mean MAE
Logit mean	chexpert	mimic_ch	rotation	0.00026	0.00067
Logit mean	chexpert	mimic_ch	vignette	0.00038	0.00044
Logit mean	mimic_ch	chexpert	blur	0.00387	0.01678
Logit mean	mimic_ch	chexpert	brightness	0.00649	0.01623
Logit mean	mimic_ch	chexpert	contrast	0.00403	0.01737
Logit mean	mimic_ch	chexpert	gamma	0.00197	0.01686
Logit mean	mimic_ch	chexpert	mixed	0.01715	0.00839
Logit mean	mimic_ch	chexpert	noise	0.02465	0.00393
Logit mean	mimic_ch	chexpert	occlusion	0.00810	0.01475
Logit mean	mimic_ch	chexpert	resolution	0.00360	0.01649
Logit mean	mimic_ch	chexpert	rotation	0.00275	0.01681
Logit mean	mimic_ch	chexpert	vignette	0.00229	0.01677
Logit RFF-MMD	chexpert	mimic_ch	blur	0.00013	0.00063
Logit RFF-MMD	chexpert	mimic_ch	brightness	0.00096	0.00084
Logit RFF-MMD	chexpert	mimic_ch	contrast	0.00062	0.00095
Logit RFF-MMD	chexpert	mimic_ch	gamma	0.00029	0.00039
Logit RFF-MMD	chexpert	mimic_ch	mixed	0.05047	0.05270
Logit RFF-MMD	chexpert	mimic_ch	noise	0.08529	0.09164
Logit RFF-MMD	chexpert	mimic_ch	occlusion	0.00250	0.00246
Logit RFF-MMD	chexpert	mimic_ch	resolution	0.00042	0.00040
Logit RFF-MMD	chexpert	mimic_ch	rotation	0.00006	0.00067
Logit RFF-MMD	chexpert	mimic_ch	vignette	0.00047	0.00044
Logit RFF-MMD	mimic_ch	chexpert	blur	0.00203	0.01678
Logit RFF-MMD	mimic_ch	chexpert	brightness	0.00239	0.01623
Logit RFF-MMD	mimic_ch	chexpert	contrast	0.00091	0.01737
Logit RFF-MMD	mimic_ch	chexpert	gamma	0.00193	0.01686
Logit RFF-MMD	mimic_ch	chexpert	mixed	0.03170	0.00839
Logit RFF-MMD	mimic_ch	chexpert	noise	0.05614	0.00393
Logit RFF-MMD	mimic_ch	chexpert	occlusion	0.02019	0.01475
Logit RFF-MMD	mimic_ch	chexpert	resolution	0.00175	0.01649
Logit RFF-MMD	mimic_ch	chexpert	rotation	0.00160	0.01681
Logit RFF-MMD	mimic_ch	chexpert	vignette	0.00126	0.01677
Probability mean	chexpert	mimic_ch	blur	0.00036	0.00063
Probability mean	chexpert	mimic_ch	brightness	0.00091	0.00084
Probability mean	chexpert	mimic_ch	contrast	0.00032	0.00095
Probability mean	chexpert	mimic_ch	gamma	0.00026	0.00039
Probability mean	chexpert	mimic_ch	mixed	0.03898	0.05270
Probability mean	chexpert	mimic_ch	noise	0.06731	0.09164
Probability mean	chexpert	mimic_ch	occlusion	0.00193	0.00246
Probability mean	chexpert	mimic_ch	resolution	0.00022	0.00040
Probability mean	chexpert	mimic_ch	rotation	0.00013	0.00067
Probability mean	chexpert	mimic_ch	vignette	0.00022	0.00044
Probability mean	mimic_ch	chexpert	blur	0.00157	0.01678
Probability mean	mimic_ch	chexpert	brightness	0.00276	0.01623
Probability mean	mimic_ch	chexpert	contrast	0.00196	0.01737
Probability mean	mimic_ch	chexpert	gamma	0.00076	0.01686
Probability mean	mimic_ch	chexpert	mixed	0.00679	0.00839
Probability mean	mimic_ch	chexpert	noise	0.00723	0.00393
Probability mean	mimic_ch	chexpert	occlusion	0.00062	0.01475
Probability mean	mimic_ch	chexpert	resolution	0.00125	0.01649

Signal	Training model	Test model	Held family	MAE	Training-family mean MAE
Probability mean	mimic_ch	chexpert	rotation	0.00156	0.01681
Probability mean	mimic_ch	chexpert	vignette	0.00060	0.01677
Probability RFF-MMD	chexpert	mimic_ch	blur	0.00015	0.00063
Probability RFF-MMD	chexpert	mimic_ch	brightness	0.00099	0.00084
Probability RFF-MMD	chexpert	mimic_ch	contrast	0.00058	0.00095
Probability RFF-MMD	chexpert	mimic_ch	gamma	0.00035	0.00039
Probability RFF-MMD	chexpert	mimic_ch	mixed	0.04976	0.05270
Probability RFF-MMD	chexpert	mimic_ch	noise	0.08585	0.09164
Probability RFF-MMD	chexpert	mimic_ch	occlusion	0.00224	0.00246
Probability RFF-MMD	chexpert	mimic_ch	resolution	0.00043	0.00040
Probability RFF-MMD	chexpert	mimic_ch	rotation	0.00008	0.00067
Probability RFF-MMD	chexpert	mimic_ch	vignette	0.00049	0.00044
Probability RFF-MMD	mimic_ch	chexpert	blur	0.00263	0.01678
Probability RFF-MMD	mimic_ch	chexpert	brightness	0.00321	0.01623
Probability RFF-MMD	mimic_ch	chexpert	contrast	0.00151	0.01737
Probability RFF-MMD	mimic_ch	chexpert	gamma	0.00127	0.01686
Probability RFF-MMD	mimic_ch	chexpert	mixed	0.03578	0.00839
Probability RFF-MMD	mimic_ch	chexpert	noise	0.04426	0.00393
Probability RFF-MMD	mimic_ch	chexpert	occlusion	0.01657	0.01475
Probability RFF-MMD	mimic_ch	chexpert	resolution	0.00164	0.01649
Probability RFF-MMD	mimic_ch	chexpert	rotation	0.00222	0.01681
Probability RFF-MMD	mimic_ch	chexpert	vignette	0.00132	0.01677
Pixel mean	chexpert	mimic_ch	blur	0.00039	0.00063
Pixel mean	chexpert	mimic_ch	brightness	0.00070	0.00084
Pixel mean	chexpert	mimic_ch	contrast	0.00086	0.00095
Pixel mean	chexpert	mimic_ch	gamma	0.00034	0.00039
Pixel mean	chexpert	mimic_ch	mixed	0.05264	0.05270
Pixel mean	chexpert	mimic_ch	noise	0.09214	0.09164
Pixel mean	chexpert	mimic_ch	occlusion	0.00244	0.00246
Pixel mean	chexpert	mimic_ch	resolution	0.00031	0.00040

Signal	Training model	Test model	Held family	MAE	Training-family mean MAE
Pixel mean	chexpert	mimic_ch	rotation	0.00089	0.00067
Pixel mean	chexpert	mimic_ch	vignette	0.00034	0.00044
Pixel mean	mimic_ch	chexpert	blur	0.02390	0.01678
Pixel mean	mimic_ch	chexpert	brightness	0.01573	0.01623
Pixel mean	mimic_ch	chexpert	contrast	0.01876	0.01737
Pixel mean	mimic_ch	chexpert	gamma	0.01447	0.01686
Pixel mean	mimic_ch	chexpert	mixed	0.00987	0.00839
Pixel mean	mimic_ch	chexpert	noise	0.00209	0.00393
Pixel mean	mimic_ch	chexpert	occlusion	0.00445	0.01475
Pixel mean	mimic_ch	chexpert	resolution	0.02357	0.01649
Pixel mean	mimic_ch	chexpert	rotation	0.01398	0.01681
Pixel mean	mimic_ch	chexpert	vignette	0.01820	0.01677
Mean error proxy	chexpert	mimic_ch	blur	0.00011	0.00063
Mean error proxy	chexpert	mimic_ch	brightness	0.00109	0.00084
Mean error proxy	chexpert	mimic_ch	contrast	0.00105	0.00095
Mean error proxy	chexpert	mimic_ch	gamma	0.00050	0.00039
Mean error proxy	chexpert	mimic_ch	mixed	0.05117	0.05270
Mean error proxy	chexpert	mimic_ch	noise	0.08959	0.09164
Mean error proxy	chexpert	mimic_ch	occlusion	0.00281	0.00246
Mean error proxy	chexpert	mimic_ch	resolution	0.00024	0.00040
Mean error proxy	chexpert	mimic_ch	rotation	0.00027	0.00067
Mean error proxy	chexpert	mimic_ch	vignette	0.00039	0.00044
Mean error proxy	mimic_ch	chexpert	blur	0.00404	0.01678
Mean error proxy	mimic_ch	chexpert	brightness	0.00143	0.01623
Mean error proxy	mimic_ch	chexpert	contrast	0.00394	0.01737
Mean error proxy	mimic_ch	chexpert	gamma	0.00208	0.01686
Mean error proxy	mimic_ch	chexpert	mixed	0.09422	0.00839
Mean error proxy	mimic_ch	chexpert	noise	0.06420	0.00393
Mean error proxy	mimic_ch	chexpert	occlusion	0.01805	0.01475
Mean error proxy	mimic_ch	chexpert	resolution	0.01080	0.01649
Mean error proxy	mimic_ch	chexpert	rotation	0.00325	0.01681
Mean error proxy	mimic_ch	chexpert	vignette	0.00398	0.01677
Error selector	chexpert	mimic_ch	blur	0.00035	0.00063
Error selector	chexpert	mimic_ch	brightness	0.00144	0.00084
Error selector	chexpert	mimic_ch	contrast	0.00135	0.00095
Error selector	chexpert	mimic_ch	gamma	0.00089	0.00039
Error selector	chexpert	mimic_ch	mixed	0.05039	0.05270
Error selector	chexpert	mimic_ch	noise	0.08722	0.09164
Error selector	chexpert	mimic_ch	occlusion	0.00273	0.00246
Error selector	chexpert	mimic_ch	resolution	0.00027	0.00040
Error selector	chexpert	mimic_ch	rotation	0.00071	0.00067
Error selector	chexpert	mimic_ch	vignette	0.00050	0.00044
Error selector	mimic_ch	chexpert	blur	0.00431	0.01678
Error selector	mimic_ch	chexpert	brightness	0.00390	0.01623
Error selector	mimic_ch	chexpert	contrast	0.00344	0.01737
Error selector	mimic_ch	chexpert	gamma	0.00285	0.01686
Error selector	mimic_ch	chexpert	mixed	0.05194	0.00839
Error selector	mimic_ch	chexpert	noise	0.05306	0.00393
Error selector	mimic_ch	chexpert	occlusion	0.02475	0.01475
Error selector	mimic_ch	chexpert	resolution	0.01206	0.01649

Signal	Training model	Test model	Held family	MAE	Training-family mean MAE
Error selector	mimic_ch	chexpert	rotation	0.00077	0.01681
Error selector	mimic_ch	chexpert	vignette	0.00626	0.01677

S6. Family-stratified cluster bootstrap

Five thousand bootstrap samples resampled three condition clusters with replacement inside every family and retained both source-model observations. Every replicate retained all held-family folds. Empirical two-sided p-values were computed for nine signals against both prespecified baselines and adjusted jointly across 18 comparisons by Holm's step-down procedure. The minimum attainable two-sided empirical value was 2/5001 = 0.0003999; floor-censored raw values and their corresponding Holm bounds are reported with "≤".

Signal	Absolute MAE, median [95% interval]	Difference from training mean, median [95% interval]	Difference from severity rank, median [95% interval]	Training mean raw/Holm p	Severity rank raw/Holm p
PCA mean	0.01442 [0.00474, 0.02524]	0.00018 [-0.00485, 0.00121]	-0.00049 [-0.00520, 0.00080]	0.8478 / 1.0000	0.5923 / 1.0000
PCA RFF-MMD	0.01543 [0.00502, 0.02712]	0.00134 [-0.00329, 0.00273]	0.00064 [-0.00368, 0.00215]	0.5071 / 1.0000	0.7011 / 1.0000
Logit mean	0.01009 [0.00399, 0.01638]	-0.00453 [-0.00896, -0.00082]	-0.00515 [-0.01023, -0.00090]	≤ 0.0004 / ≤ 0.0072	≤ 0.0004 / ≤ 0.0072
Logit RFF-MMD	0.01230 [0.00448, 0.02120]	-0.00226 [-0.00499, -0.00010]	-0.00279 [-0.00611, -0.00017]	0.0280 / 0.3079	0.0232 / 0.2783
Probability mean	0.00674 [0.00257, 0.01101]	-0.00798 [-0.01411, -0.00228]	-0.00837 [-0.01538, -0.00237]	≤ 0.0004 / ≤ 0.0072	≤ 0.0004 / ≤ 0.0072
Probability RFF-MMD	0.01195 [0.00413, 0.02047]	-0.00273 [-0.00573, -0.00050]	-0.00317 [-0.00701, -0.00060]	≤ 0.0004 / ≤ 0.0072	≤ 0.0004 / ≤ 0.0072
Pixel mean	0.01527 [0.00539, 0.02559]	0.00050 [-0.00005, 0.00283]	0.00011 [-0.00205, 0.00264]	0.1152 / 1.0000	0.8914 / 1.0000
Mean error proxy	0.01693 [0.00504, 0.03117]	0.00265 [-0.00244, 0.00639]	0.00195 [-0.00283, 0.00562]	0.4371 / 1.0000	0.5163 / 1.0000
Error selector	0.01472 [0.00506, 0.02532]	0.00026 [-0.00259, 0.00185]	-0.00033 [-0.00310, 0.00141]	0.8026 / 1.0000	0.7542 / 1.0000

Training-mean baseline absolute MAE: 0.01475 [0.00480, 0.02494]. Severity-rank baseline absolute MAE: 0.01511 [0.00490, 0.02647].

On the expanded 30-condition panel, condition-level Spearman correlation with paired Brier harm was 0.650 for PCA-representation mean displacement and 0.642 for PCA-representation RFF-MMD. Their orderings are therefore not identical; the identical ordering seen in the smaller pilot grid was a grid-specific observation, not a general property.

S7. Descriptive outcome-robustness audit

All five outcomes below were prespecified, but only the primary Brier outcome was prespecified for the held-source-domain/held-family transfer and multiplicity-controlled superiority analysis. As a post hoc audit, the same 20 folds, signal standardization, ridge penalty, and training-family mean-harm comparator were rerun separately for the four secondary outcomes. The primary MAEs reproduced exactly (maximum absolute difference 0.0e+00). These secondary ratios and associations are descriptive checks; no new p-values or superiority claims are made.

For macro AUC and locked F1, change was multiplied by -1 so larger values consistently denote more harm. A transfer ratio below 1 denotes lower MAE than the outcome-specific training-family mean-harm baseline.

Signal	Brier	Log loss	ECE	Macro-AUC loss	Locked-F1 loss
PCA mean	1.038	0.982	0.936	0.603	0.679
PCA RFF-MMD	1.112	1.054	1.030	0.570	0.609
Logit mean	0.705	0.621	0.629	0.931	1.094
Logit RFF-MMD	0.884	0.820	0.815	0.586	0.768
Probability mean	0.459	0.329	0.301	1.630	1.740
Probability RFF-MMD	0.850	0.782	0.775	0.585	0.748
Pixel mean	1.002	1.018	1.017	0.917	0.934
Mean error proxy	1.195	1.154	1.150	0.760	0.777
Error selector	1.046	1.011	1.010	0.697	0.797

Table S7.1. Held-source-domain/held-family MAE ratio relative to the training-family mean-harm baseline.

Signal	Brier	Log loss	ECE	Macro-AUC loss	Locked-F1 loss
PCA mean	0.513	0.550	0.539	0.661	0.480
PCA RFF-MMD	0.400	0.436	0.463	0.666	0.572
Logit mean	0.876	0.896	0.917	0.530	0.365
Logit RFF-MMD	0.690	0.724	0.711	0.695	0.493
Probability mean	0.969	0.972	0.994	0.399	0.246
Probability RFF-MMD	0.721	0.753	0.759	0.660	0.487
Pixel mean	0.007	0.005	0.012	0.131	0.200
Mean error proxy	0.254	0.276	0.322	0.393	0.359
Error selector	0.490	0.515	0.556	0.531	0.433

Table S7.2. Descriptive Pearson R^2 between each signal and positively oriented harm across the 60 model-condition rows. These in-panel associations do not use held-family prediction and should not be interpreted as external calibration.

Signal	Brier	Log loss	ECE	Macro-AUC loss	Locked-F1 loss
PCA mean	0.650	0.693	0.677	0.542	0.596
PCA RFF-MMD	0.642	0.688	0.669	0.544	0.598
Logit mean	0.671	0.729	0.465	0.630	0.674
Logit RFF-MMD	0.713	0.776	0.518	0.700	0.743
Probability mean	0.742	0.788	0.517	0.681	0.751
Probability RFF-MMD	0.725	0.789	0.497	0.700	0.768
Pixel mean	0.217	0.306	0.064	0.377	0.338
Mean error proxy	0.421	0.374	0.314	0.372	0.379
Error selector	0.403	0.370	0.287	0.378	0.344

Table S7.3. Descriptive Spearman correlation with positively oriented harm. Probability mean displacement retained positive correlations of 0.517–0.788 across the five outcomes, even where its held-family magnitude ratios exceeded 1. This separates condition ordering from calibrated magnitude transfer. The high probability-mean/Brier R^2 (0.969) is consistent with functional overlap because both use model probabilities, but it is not an algebraic identity: Brier change also includes observed labels and residual cross-terms.

The probability signal and Brier outcome both use the same frozen per-label logistic calibration maps. A post hoc descriptive sensitivity analysis removed those maps from both quantities and used raw sigmoid outputs while retaining the same 60 model-condition rows and 20 held-source-domain/held-family folds. The reconstructed calibrated Brier changes matched the primary values to a maximum absolute error of 5.55e-17.

Probability construction	Brier held-family MAE ratio	Spearman with Brier harm	Pearson R^2
Frozen logistic calibrators (submitted)	0.459	0.742	0.969
Raw sigmoid; calibrators removed	0.440	0.367	0.957

Table S7.4. Post hoc calibration-removal sensitivity. The held-family ratio changed little, from 0.459 to 0.440, whereas the in-panel rank association was sensitive to the processing choice. This descriptive audit adds no p-value or superiority claim.

ECE increased (worsened) in 38/60 model-condition rows and decreased (improved) in 22/60. Signed ECE change, rather than absolute displacement, is used throughout.

S8. Error-estimator and selector calibration

For each source model, 2000 calibration images trained the gradient-boosting error regressor and 2000 different calibration images selected the quantile pair. The grid searched true-error quantiles 0.50–0.95 and predicted-error quantiles 0.10–0.90. The chosen eligible pair maximized selector power subject to false-discovery proportion <= 0.20; if no pair was eligible, the minimum-FDP pair was retained as a negative comparator. Both models yielded an eligible quantile pair. Cross-validated R^2 values were 0.058 and 0.081, so this is a weak batch analogue rather than a full-strength evaluation of the Amoukou method. No monitor label contributed to fitting or selection.

S9. Clean-versus-clean tests

Thirty 200/200 clean reference resamples estimated empirical type-I behavior separately for PCA-representation and logit RFF-MMD. The p-value calculation used 999 label permutations and (1 + exceedances)/(1 + 999). CheXpert-trained PCA/logit monitoring each produced 1/30 false positives; MIMIC-CXR-trained PCA/logit monitoring each produced 2/30. The corresponding proportions 0.033 and 0.067 are reported with their small denominators rather than interpreted as evidence of type-I miscalibration.

A separate post hoc descriptive diagnostic tested clean validation-reference against clean test-monitor pools, matching the deployment-style comparison used for the 30 image conditions. It reused the exact PCA and RFF maps already constructed by the confirmatory analysis. A dedicated seed stream prevented the new permutations from changing any frozen condition test or bootstrap result. Removing only the diagnostic block made every pre-existing analysis field exactly equal; the condition-level CSV remained byte-identical.

Model	Space	Alarms/30	Rate	Exact binomial 95% CI	Median p
chexpert	PCA representation	2/30	0.067	[0.008, 0.221]	0.415
chexpert	logit	2/30	0.067	[0.008, 0.221]	0.363
mimic ch	PCA representation	1/30	0.033	[0.001, 0.172]	0.372
mimic ch	logit	2/30	0.067	[0.008, 0.221]	0.535

Table S9.1. Post hoc clean validation-reference versus clean test-monitor diagnostic. The four median p-values are 0.363–0.535 and the alarm counts are 1–2/30, providing no evidence of a systematic validation-to-test boundary alarm.

S10. Post hoc output-scope, task-subspace, and PCA-direction audits

The frozen confirmatory task-null control constrained the five outputs analyzed in the article. A later scope audit examined the complete released classifier layer. Each checkpoint exposes 18 output slots. Seven—Infiltration, Emphysema, Fibrosis, Pleural Thickening, Nodule, Mass, Hernia—have denormal-scale classifier rows with maximum float64 norm 2.58e-38 and are numerically inactive at operating precision; the 18 × 1024 matrix has numerical rank 11 and a 1013-dimensional null space. The whole-model-null extension projects the same leading-PC source into this complete null space and therefore preserves every numeric output slot.

Model	Released output slots	Classifier rank	Whole-model null dimension	Effective rank of five selected logits	Five selected-logit variance shares
chexpert	18	11	1013	1.13	0.941, 0.032, 0.019, 0.007, 0.002
mimic ch	18	11	1013	1.23	0.901, 0.056, 0.026, 0.014, 0.003

Table S10.1. Exact released-head scope and reference-logit geometry. Effective rank is the participation ratio of the five selected-logit covariance spectrum. The first component accounts for approximately 94% and 90% of selected-logit variance, but this concentration alone does not establish why the five-logit and PCA alarm rates differ.

Model	Whole-model-null magnitude	Maximum absolute change across 18 slots	Paired Brier harm	PCA RFF-MMD	Five-logit RFF-MMD	PCA p	Five-logit p
chexpert	0.25	5.329e-15	-0.00000000	0.0568475	0.0000000	0.001	0.689
chexpert	0.50	1.066e-14	-0.00000000	0.2236596	0.0000000	0.001	0.810
chexpert	1.00	8.882e-15	-0.00000000	0.7416193	0.0000000	0.001	0.165
mimic ch	0.25	1.421e-14	-0.00000000	0.0421300	0.0000000	0.001	0.365
mimic ch	0.50	2.309e-14	-0.00000000	0.1667199	0.0000000	0.001	0.875
mimic ch	1.00	4.086e-14	-0.00000000	0.5488241	0.0000000	0.001	0.844

Table S10.2. Post hoc whole-model-null control. All six PCA tests alarmed at p = 0.001; no five-logit test alarmed. The maximum absolute float64 change across every released output slot was 4.086e-14. Dedicated permutation streams leave all frozen results unchanged.

The original five-output-null control preserves the analyzed logits but can move other active released outputs; Table S10.3 quantifies this narrower scope. Only the whole-model-null extension supports the article's all-output invariance claim.

Model	Magnitude	Maximum selected-output change	Largest excluded absolute-change output	Absolute change	Largest excluded standardized-change output	Change in reference SD units
chexpert	0.25	8.049e-16	Pneumonia	0.548	Pneumothorax	0.37
chexpert	0.50	1.610e-15	Pneumonia	1.096	Pneumothorax	0.75
chexpert	1.00	3.220e-15	Pneumonia	2.192	Pneumothorax	1.49
mimic ch	0.25	4.406e-16	Enlarged Cardiomediastinum	0.374	Lung Lesion	0.18
mimic ch	0.50	8.812e-16	Enlarged Cardiomediastinum	0.747	Lung Lesion	0.35
mimic ch	1.00	1.762e-15	Enlarged Cardiomediastinum	1.494	Lung Lesion	0.70

Table S10.3. Output-scope audit of the frozen five-output-null control. Standardization uses the reference-logit spread and includes every active nonselected output; numerically inactive rows have no meaningful output variation. Task-nullity is relative to the output set used in the projection.

The exact released 5 × 1024 heads—not least-squares reconstructions—defined $P = W^T(WW^T)^+W$. For an orientation-free isotropic displacement in 1024 dimensions, the expected squared-energy fraction in this rank-5 row space is 5/1024 = 0.00488. Table S10.4 reports projection of mean paired feature change and summed paired-sample energy ratios together with enrichment over that geometric benchmark; these are Euclidean feature energies, not MMD, explained harm, or causal mediation.

Model	Displacement summary	Median task-row fraction	Range across conditions	Enrichment over 5/1024
chexpert	Paired mean displacement	0.078	0.029–0.333	16.0×
chexpert	Summed paired per-image energy	0.135	0.059–0.213	27.7×
mimic ch	Paired mean displacement	0.117	0.034–0.229	24.1×
mimic ch	Summed paired per-image energy	0.126	0.066–0.166	25.8×

Table S10.4. Exact-head task-row fractions over 30 conditions. Real perturbations were strongly enriched toward the five-output task row space relative to an orientation-free displacement, yet the paired-sample complement—approximately 86–87% at the median condition—was squared feature-change energy in null(W) and made no linear contribution to the five selected logit changes. Both observations are required: enrichment can make broad shift severity co-vary with harm, whereas the larger task-null share prevents total representation change from being interpreted as task harm.

The component norms were also passed through the already submitted held-source-domain/held-family mapping as a descriptive mechanism check. They were not added to the nine-signal confirmatory family and receive no p-values or superiority claims. The 16.0–27.7-fold row-space enrichment helps explain why task-row change, task-null change, and harm can all rise with ordered corruption severity. Similar task-row and task-null Brier ratios nevertheless show why predictive association is not causal attribution; for AUC loss, the null component can be a better severity proxy despite making no direct logit contribution.

Post hoc component signal	Brier transfer ratio	Macro-AUC-loss transfer ratio
Deployment-reference task-row mean norm	0.822	0.723
Deployment-reference task-null mean norm	0.845	0.662
Paired-clean task-row mean norm	0.842	0.725
Paired-clean task-null mean norm	0.847	0.656

Table S10.5. Descriptive MAE ratios relative to the outcome-specific training-family mean-harm baseline.

The submitted exact control used the leading reference PC after removal of its task-row component. To test whether that choice was uniquely favorable, a dedicated-RNG audit repeated the intervention along PCs 8, 32, and 64 and a random task-null direction. It also constructed a direction in the exact intersection null(W) ∩ null(PCA64). All frozen analysis fields and the condition CSV remained exactly unchanged. The retained and random directions were detected at p = 0.001 in all 30 model/direction/magnitude tests. The outside-span direction was not detected in any of six tests (p = 0.559–0.866), as required by the 64-component truncation.

Model	Task-null direction	Energy retained by PCA64	Maximum absolute logit change	p at 0.25	p at 0.50	p at 1.00
chexpert	outside retained pca span	0.0000	4.01e-15	0.660	0.559	0.866
chexpert	pc 1 submitted	0.7909	3.44e-15	0.001	0.001	0.001
chexpert	pc 32	0.9924	1.33e-15	0.001	0.001	0.001
chexpert	pc 64	0.9950	5.55e-16	0.001	0.001	0.001
chexpert	pc 8	0.9074	2.55e-15	0.001	0.001	0.001
chexpert	random unit	0.0728	4.02e-16	0.001	0.001	0.001
mimic ch	outside retained pca span	0.0000	1.68e-15	0.756	0.866	0.814
mimic ch	pc 1 submitted	0.7661	1.61e-15	0.001	0.001	0.001

Model	Task-null direction	Energy retained by PCA64	Maximum absolute logit change	p at 0.25	p at 0.50	p at 1.00
mimic ch	pc 32	0.9992	5.55e-16	0.001	0.001	0.001
mimic ch	pc 64	0.9853	1.44e-15	0.001	0.001	0.001
mimic ch	pc 8	0.9433	1.67e-15	0.001	0.001	0.001
mimic ch	random unit	0.0565	4.25e-16	0.001	0.001	0.001

Table S10.6. Descriptive direction-robustness audit using the exact submitted PCA/RFF transforms and isolated permutation streams. Detection is robust across the sampled directions visible to PCA64, while the exact outside-span construction documents the monitor's blind spot. These directions are null for the five selected outputs; the whole-model extension above is the output-scope-robust control.

The machine-readable condition-level mechanism results are supplied in results/task-subspace-audit.csv and results/task-subspace-audit.json.

S11. Extraction cache manifest

Role or condition	Extraction seconds	SHA-256
calibration-clean	0.0	e74082a55887375ca927fca350fd0d71d30238ec96cd0c8c0746b95da815897f
reference-clean	0.0	2c5220339afddd9487a26f9a919d80e26509e411b9310ca90203e5c1c640e8b2
monitor-clean	0.0	a4179714d1b82e33a75a015be548ebd82b47f9ade3490c8c32366e0e3c2dde03
brightness_0.03	0.0	7f2582b9fd026d4400552a4191915eedec1230914170b93ed86a3a15daecf7a1
brightness_0.06	0.0	b0ea568117277ee8fe9fa385e8281e64b1088238ff05f5c1e442f608a46945cf
brightness_0.12	0.0	66ed5b4586d2b38550b3b33b129e2d6ae21e01e1ffacb748fe8e34067e0b72ef
contrast_0.15	0.0	660b48776a7853141ffc75aa2099b1574c2a4f0d0384f0d15c4b7b76fb9dfbf6
contrast_0.3	0.0	ad9befb6513e835f57c8be1acae9a553e11a89e765c0e7e7089f7973f2530471
contrast_0.5	0.0	b0f6a6c331b611b70aaca3389443263f6fbb179893e46cd8a32dd5d1d4690c64
gamma_1.15	0.0	41acf5bf8f9a9dd91976ac1ae9e7794b2a8b65ce12a8246cfdbc401728d68c2f

Role or condition	Extraction seconds	SHA-256
gamma_1.35	0.0	4534fe6401e4922558940b036543d77cad-fed3bab57736938ec0a368ee687090
gamma_1.6	0.0	9362c1471c6acb31411c6536205b228dc27fb61cdb4988852e13c2a849f3acc3
noise_0.02	0.0	7c19b7ee8d2cd85317194d0f5af815cc20cf6308f94a5654fe3968176e788e1f
noise_0.05	0.0	20231b400fa1328edbf517b18b3005f4c6fbda8c384a51187c27c446e5181797
noise_0.1	0.0	df45effc0269acda552b8b99006224f7e3c68e1c1722c6f979987ca25a678be8
blur_0.25	0.0	15864c0ad315d37b5674421aacb83cf828f634d7325843ca774f84990da11e15
blur_0.5	503.6	449c0504b3e85fe2df7ca95fa6d075b30bbb118c263a7fba37bf6e0de30d5751
blur_0.8	492.9	e2d5f134edb86ef7b45d4c790926c27e70861592855d7caa97dc5022c84fb94a
rotation_3	509.0	8be29438d678e0e9004876954ff58d09fb0fe91ae84c0efb1ba62aaae544bd82
rotation_7	512.3	5bdf219f9144c3c015141f751e5c3c3065c223d3be8e43942a0d3d31ddf3b36d
rotation_12	155.9	f85e6c30b3a82dbdfd17614bd309b3b964fb5ffbef1226127145b5f3760638a7
resolution_0.75	201.6	7642f7428d8a45493add3f673073fa419d79181fe95406dd4298a3ff72e06eed
resolution_0.5	331.4	057e2d2d44527ec7e9633ae012255706d96b3e1282df19524284a04443c1deaa
resolution_0.35	204.9	4455503ca1999543b4ce3f6b5b6b237dc23eb24bf9c20d940312a86f9584b837
occlusion_0.05	185.6	c1c1120945125a1bca948e0d42cac7c91e55b9c1fd9db04bc721e9b54755c07f
occlusion_0.1	148.1	777ed766b579fd217f375a40b61b3a61d50cf96b70fea6745ea1cc4c71531597
occlusion_0.2	150.5	159ba1642d49a2abe3c0f60c58b9c7db907c7fa6a4a63ad9b5321e49485f34ba

Role or condition	Extraction seconds	SHA-256
vignette_0.1	163.1	6d3d92f78f425751d9ba26be692f0f2eff95b09dcf8344682f8fdf77b3ee79da
vignette_0.25	160.8	215d4bf51ea21ccf2e3c65ce3a343281a1321dfcb0e2f456de6f6be4a7f93f94
vignette_0.4	163.6	620730da8ff8e3dce66e8029c847ce71e92719a8de5f95e3bfd72b6798bc545d
mixed_1	161.1	0b1458627259527f8ec5aed2f63cc028ac3ea8d327842f61d5860fbc95e89703
mixed_2	161.0	6bf72c56b73c60e412dd84889089cea2309f5420adafed93d3bf676436bb412c
mixed_3	159.1	1dd1685dc7733308b6bfee14742afc0500ec51b20101dcf0dae70e1034456d04

S12. Reproduction order

1. Run code/01_validate_chexpert_model.py once for each public weight identifier.
2. Run code/02_extract_confirmatory.py with the frozen default role sizes and all conditions.
3. Run code/03_analyze_confirmatory.py with 5000 bootstrap replicates.
4. Run code/12_analyze_outcome_robustness.py to reproduce the post-analysis descriptive outcome audit.
5. Run code/13_analyze_task_subspace.py to reproduce the post hoc exact-head mechanism audit.
6. Run code/18_analyze_review_sensitivity.py to reproduce the post hoc dimension, threshold, severity, signal-type, and resource audits.
7. Run code/architecture/01_extract_architecture_features.py and code/architecture/02_analyze_architecture_sensitivity.py to reproduce the exploratory architecture arm; run code/architecture/03_verify_architecture_experiment.py to check hashes, coverage, p-value grids, and exact-control tolerances.
8. Run code/20_make_reviewer_readable_figures.py to regenerate Figures 1–3; use the existing figure generator for Figures 4–5 and the sensitivity script for Figure 6.
9. Run the manuscript and supplement document builders.
10. Run code/14_make_graphical_abstract.py to regenerate the optional portal graphical abstract.

S13. Shift generation and output-slot scope

S13.1. Exact image-transform parameters

All transforms operated on tensors scaled to [0, 1] and were clipped back to that interval where required. Low, medium, and high denote parameter order only; they are not clinical severity grades.

Family	Low	Medium	High	Implementation
Brightness	+0.03	+0.06	+0.12	Add a constant intensity offset
Contrast	+0.15	+0.30	+0.50	Multiply deviations from the reference mean by 1+s
Gamma	1.15	1.35	1.60	Power-law intensity transform
Gaussian noise	SD 0.02	SD 0.05	SD 0.10	Add independent zero-mean Gaussian noise
Blur	0.25	0.50	0.80	Blend original with 3 x 3 average-filtered image
Rotation	3 degrees	7 degrees	12 degrees	In-plane rotation using the frozen interpolation settings

Family	Low	Medium	High	Implementation
Resolution	75%	50%	35%	Downsample to the retained fraction and restore to 224 x 224
Central occlusion	5%	10%	20%	Mask a central square occupying the stated fraction of image area
Vignette	0.10	0.25	0.40	Apply radial attenuation with stated maximum strength
Mixed	0.02 / 0.20 / 0.01	0.05 / 0.40 / 0.03	0.09 / 0.65 / 0.06	Brightness / blur blend / noise SD

The transform panel intentionally separates visually and statistically different perturbation axes. It is a reproducible stress test and is not asserted to simulate a specific hospital, scanner, demographic transition, temporal drift, or prevalence shift.

S13.2. Released classifier slots and five-label outcome panel

The two frozen TorchXRayVision identifiers were densenet121-res224-chex and densenet121-res224-mimic_ch. Both use a 1024-dimensional penultimate representation and expose 18 classifier slots. This study did not retrain or fine-tune them.

Slot group	Labels	Role in this study
Prespecified outcome panel (5)	Atelectasis; Cardiomegaly; Effusion; Consolidation; Edema	Calibration, thresholding, harm outcomes, and selected-head controls
Active and shared with ChestMNIST, outside panel (2)	Pneumonia; Pneumothorax	Not used for harm outcomes; included in whole-model invariance audit
Active source outputs absent from ChestMNIST label set (4)	Lung Lesion; Fracture; Lung Opacity; Enlarged Cardiomediastinum	No target outcome available; included in whole-model invariance audit
Numerically inactive rows (7)	Infiltration; Emphysema; Fibrosis; Pleural Thickening; Nodule; Mass; Hernia	Denormal-scale head rows; included in the 18-slot audit

The five-label panel was fixed before shifted outcomes were extracted and was not chosen using condition results. It is nevertheless an analyst-defined scope. A different active-label panel could change metric-specific harm and signal rankings. The 18-slot whole-model-null control resolves only whether the released linear head changes under the exact intervention.

S14. Sensitivity and resource-profile audit

All analyses in this section are post hoc and descriptive. Dedicated random-number streams and fixed RFF maps were used so these runs did not change the confirmatory permutation stream or multiplicity analysis.

S14.1. Retained PCA dimension

Table S14.1. Alarm rate and held-family Brier-harm MAE ratio by retained PCA dimension. Ratios are relative to the training-family mean-harm baseline; values below 1 favor the signal descriptively.

PCA components	PCA alarm rate	PCA mean-displacement ratio	PCA RFF-MMD ratio
16	0.917	0.964	1.002
32	0.917	1.058	1.116
64	0.933	1.038	1.145
128	0.933	1.216	1.169
256	0.983	1.362	1.135

Higher dimension increased detectability but did not create a stable harm-prediction advantage. PCA was refitted and a dedicated RFF realization with 499 permutations was used at each dimension. The 64-component audit rate (0.933) is therefore a sensitivity rerun, not an exact reproduction of the frozen 64-component, 999-permutation rate (0.967); the audit answers the sensitivity question without changing the confirmatory result.

S14.2. Alarm threshold and severity

Table S14.2. Alarm rates from the original 999-permutation p-values.

Signal	Alpha	Overall	Low	Medium	High
PCA RFF-MMD	0.01	0.900	0.800	0.950	0.950
PCA RFF-MMD	0.05	0.967	0.900	1.000	1.000
PCA RFF-MMD	0.10	0.967	0.900	1.000	1.000
Five-logit RFF-MMD	0.01	0.417	0.250	0.500	0.500
Five-logit RFF-MMD	0.05	0.517	0.450	0.550	0.550
Five-logit RFF-MMD	0.10	0.533	0.450	0.600	0.550

The gap between PCA and logit detection persists across thresholds. Both monitors, especially the logit monitor, have less power at low parameter levels.

S14.3. Incremental computation and memory

The profile covers signal calculation for one 200-case window after images have been decoded and the CNN has produced representations and outputs. Each signal received 15 warm-up calls and 200 timed repetitions. Persistent state includes fitted projections, RFF parameters, or the error model; working memory is the approximate footprint of arrays materialized for a window. Values are means across the two checkpoints and should not be interpreted as end-to-end deployment latency.

Signal	Median latency (ms)	Persistent state (KiB)	Working arrays (KiB)
Logit mean displacement	0.019	0.078	7.81
Raw-pixel projection mean displacement	0.224	1.50	100.0
PCA mean displacement	0.766	520.5	100.0
Logit RFF-MMD	0.968	13.08	207.8
Probability mean displacement	0.949	0.195	7.81
Probability RFF-MMD	2.043	13.20	207.8
PCA RFF-MMD	2.195	651.0	300.0
Mean learned-error proxy	5.793	474.0	39.1
Calibrated high-error selector	6.075	474.0	39.1

S14.4. Exploratory modern-architecture comparison

S14.4.1. Frozen exploratory design

This arm was specified separately after the primary DenseNet-121 results and is not pooled with the confirmatory estimand. The protocol file was frozen before the two additional weight sets were downloaded or any architecture-arm outcome was analysed. It compared:

- convnextv2_atto.fcmae_ft_in1k, a pure convolutional ConvNeXt V2 encoder with a 320-dimensional pooled representation and 224 × 224 input; and
- mobilevit_xs.cvnets_in1k, a convolution-transformer hybrid encoder with a 384-dimensional pooled representation and 256 × 256 input.

Both public ImageNet-pretrained encoders were loaded through timm 1.0.28 and frozen. Their state hashes were 46633ca3ccdfb0e97199ce30d5707626a558f65a7fe3c7ba83bb89185a261785 and 852996534db93b8c6d8210a3f80152ade7d5d1a2de076548fc960962fed436ce, respectively. Images were transformed in the original 224-pixel grayscale space, then repeated to three channels, deterministically resized when required, and normalized according to the released model configuration.

For each architecture and label, an L2-regularized logistic head (C=1, lbfgs, maximum 2000 iterations) was fitted on all 78,468 official training images. The resulting five-row heads had rank 5. The original 4000 calibration, 2000 reference, and 2000 monitor indices and labels were reused exactly. Calibrators, F1 thresholds, 30 shifts, signal implementations, error-proxy calibration, 999-

permutation alarm tests, paired outcomes, and exact controls followed the primary analysis. This matched design deliberately held the cohort, labels, role indices, head class and training procedure, shifts, and estimands fixed so that encoder family was the principal changed factor rather than being confounded with a different clinical task or dataset. The quality gate required macro AUC ≥ 0.70 and at least four label AUCs ≥ 0.65 on the untouched clean monitor role.

S14.4.2. Clean-monitor quality and calibration

Both models passed the gate, and all five per-label AUCs exceeded 0.65. Thresholds were selected only on the calibration role and then locked.

Model	Label	Positives/2000	AUC	Locked F1	Brier	Threshold
ConvNeXtV2-Atto	Atelectasis	198	0.724	0.283	0.08451	0.15
ConvNeXtV2-Atto	Cardiomegaly	64	0.787	0.174	0.02990	0.08
ConvNeXtV2-Atto	Effusion	273	0.775	0.415	0.10656	0.16
ConvNeXtV2-Atto	Consolidation	95	0.758	0.239	0.04288	0.09
ConvNeXtV2-Atto	Edema	29	0.781	0.000	0.01439	0.13
MobileViT-XS	Atelectasis	198	0.742	0.307	0.08417	0.16
MobileViT-XS	Cardiomegaly	64	0.732	0.131	0.03025	0.06
MobileViT-XS	Effusion	273	0.783	0.403	0.10331	0.22
MobileViT-XS	Consolidation	95	0.770	0.220	0.04296	0.10
MobileViT-XS	Edema	29	0.832	0.114	0.01384	0.10

Table S14.3. Per-label clean-monitor quality of the two exploratory heads. Macro AUC/F1/mean Brier were 0.765/0.222/0.05565 for ConvNeXtV2-Atto and 0.772/0.235/0.05491 for MobileViT-XS. The zero edema F1 for ConvNeXtV2-Atto reflects the locked calibration-role threshold and low monitor prevalence; it was not retuned.

S14.4.3. Alarm behavior and clean diagnostics

Model	Image-condition representation alarms	Image-condition logit alarms	Clean-boundary representation alarms	Clean-boundary logit alarms	Median clean-boundary representation p	Median clean-boundary logit p
ConvNeXtV2-Atto	28/30	20/30	1/30	2/30	0.4435	0.4275
MobileViT-XS	29/30	16/30	0/30	2/30	0.4775	0.4205

Table S14.4. RFF-MMD alarms at alpha = 0.05. Clean-boundary episodes compare validation-reference with clean-test monitor windows; they use independent random-number streams and do not alter condition tests.

Across the 30 image conditions, paired Brier harm ranged from −0.00038 to 0.01262 for ConvNeXtV2-Atto and from −0.00025 to 0.00827 for MobileViT-XS; harm was positive in 24/30 and 19/30 conditions. Median harms were 0.00060 and 0.00039. The complete 60-row condition file is supplied as results/architecture-condition-results.csv.

S14.4.4. Exact task-null and task-aligned controls

Model	Control	Magnitude	Maximum logit/target error	Representation p	Logit p	Paired Brier harm
ConvNeXtV2-Atto	Task-null	0.25	4.44e-15	0.029	0.874	0.00000000
ConvNeXtV2-Atto	Task-null	0.50	4.44e-15	0.001	0.548	0.00000000
ConvNeXtV2-Atto	Task-null	1.00	6.22e-15	0.001	0.700	0.00000000
ConvNeXtV2-Atto	Task-aligned	0.25	3.55e-15	0.970	0.097	0.00024459
ConvNeXtV2-Atto	Task-aligned	0.50	3.55e-15	0.694	0.001	0.00139013
ConvNeXtV2-Atto	Task-aligned	1.00	4.44e-15	0.294	0.001	0.00777775
MobileViT-XS	Task-null	0.25	6.66e-15	0.032	0.714	1.39e-17
MobileViT-XS	Task-null	0.50	7.99e-15	0.001	0.896	0.00000000
MobileViT-XS	Task-null	1.00	9.77e-15	0.001	0.116	0.00000000
MobileViT-XS	Task-aligned	0.25	6.66e-15	0.463	0.903	0.00028440
MobileViT-XS	Task-aligned	0.50	7.99e-15	0.256	0.001	0.00151523
MobileViT-XS	Task-aligned	1.00	7.11e-15	0.520	0.001	0.00820861

Table S14.5. Exact-control audit. “Maximum logit/target error” is the maximum absolute logit change for task-null controls and the maximum error from the requested common logit offset for task-aligned controls. All six task-null representation tests alarmed, no task-null logit test alarmed, and paired Brier harm was zero to numerical tolerance. At magnitudes 0.50 and 1.00, all four task-aligned logit tests alarmed while no task-aligned representation test alarmed. This is a construct reversal, not a claim that one space is uniformly more powerful.

S14.4.5. Held-architecture and held-family Brier-harm transfer

Signal	Mean MAE	Training-mean MAE	Ratio	Folds improved/20	Spearman rho with harm
Probability mean displacement	0.000874	0.002164	0.404	18	0.867
Probability RFF-MMD	0.001012	0.002164	0.468	19	0.872
Logit mean displacement	0.001093	0.002164	0.505	16	0.868
PCA-representation mean displacement	0.001169	0.002164	0.540	16	0.853
Logit RFF-MMD	0.001183	0.002164	0.546	19	0.848
PCA-representation RFF-MMD	0.001272	0.002164	0.588	18	0.830
Calibrated high-error selector	0.001637	0.002164	0.757	16	0.550
Mean learned-error proxy	0.001837	0.002164	0.849	14	0.491
Raw-pixel projection mean displacement	0.002415	0.002164	1.116	6	−0.139
Ordinal severity rank baseline	0.002181	0.002164	1.008	4	—

Table S14.6. Descriptive bidirectional architecture/family transfer. Each of 20 folds trains on nine families from one architecture and tests the held family in the other architecture, then reverses

direction. Ratios below 1 improve on the source-training-family mean-harm predictor. No multiplicity-adjusted superiority inference was added for this exploratory arm.

The error proxies remained weak (cross-validated R^2 0.041 and 0.067) but both calibrated selectors met the FDP $\leq$ 0.20 constraint, with FDP 0.173/0.164 and power 0.827/0.836. This matches the primary arm's caution that selector performance depends on the quality of the upstream error model.

S14.4.6. Integrity and evidence boundary

The independent audit verified 102 cached files by SHA-256, all 60 architecture-condition rows, the 999-permutation p-value grid, the two quality-gate decisions, and exact-control reconstruction. Maximum task-null logit changes were 6.22×10^{-15} and 9.77×10^{-15}; maximum task-aligned target errors were 4.44×10^{-15} and 7.99×10^{-15}. The audit reported zero errors. Model weights and large derived feature caches are not redistributed; the state hashes, fitted linear-head coefficients, role indices, scripts, compact condition results, and integrity report are supplied.

This arm is a controlled architecture replication: with the cohort, labels, role indices, head training, shifts, and analysis held fixed, the exact separation between broad representation detectability and head-mediated harm was reproduced in a modern convolutional encoder and a convolution-transformer hybrid. Both heads achieved macro AUC above 0.75 and passed every per-label AUC floor, reducing the concern that the qualitative construct result is unique to the sub-threshold DenseNet checkpoints. The matched controls strengthen attribution to encoder-family change but do not establish clinical-system generalization because both heads were trained on ChestMNIST, both encoders began from ImageNet pretraining, all comparisons use one benchmark cohort and algorithmic perturbation panel, and the arm was specified after the primary analysis. Prospective confirmation requires architectures, training domains, quality gates, and clinical streams fixed before outcomes are observed.
